# New Eudesmane-Type Sesquiterpenoids from the Mangrove-Derived Endophytic Fungus *Penicillium* sp. J-54

**DOI:** 10.3390/md16040108

**Published:** 2018-03-28

**Authors:** Liuming Qiu, Pei Wang, Ge Liao, Yanbo Zeng, Caihong Cai, Fandong Kong, Zhikai Guo, Peter Proksch, Haofu Dai, Wenli Mei

**Affiliations:** 1 Key Laboratory of Biology and Genetic Resources of Tropical Crops, Ministry of Agriculture, Institute of Tropical Bioscience and Biotechnology, Chinese Academy of Tropical Agricultural Sciences, Haikou 571101, China; qiulm520@163.com (L.Q.); wangpei@itbb.org.cn (P.W.); geliao828@gmail.com (G.L.); zengyanbo@itbb.org.cn (Y.Z.); caicaihong@itbb.org.cn (C.C.); kongfandong@itbb.org.cn (F.K.); guozhikai@itbb.org.cn (Z.G.); 2 Liuzhou Railway Secondary Middle School, Heping Road, Liuzhou 545007, Guang Xi, China; 3 Institute of Pharmaceutical Biology and Biotechnology, Heinrich-Heine University Duesseldorf, 40225 Duesseldorf, Germany; proksch@uni-duesseldorf.de

**Keywords:** endophytic fungus, *Penicillium* sp., sesquiterpenoids, cytotoxicity

## Abstract

Four new eudesmane-type sesquiterpenoids, penicieudesmol A–D (**1**–**4**), were isolated from the fermentation broth of the mangrove-derived endophytic fungus *Penicillium* sp. J-54. Their structures were determined by spectroscopic methods, the in situ dimolybdenum CD method, and modified Mosher’s method. The bioassays results showed that **2** exhibited weak cytotoxicity against K-562 cells.

## 1. Introduction

**Figure 1:**
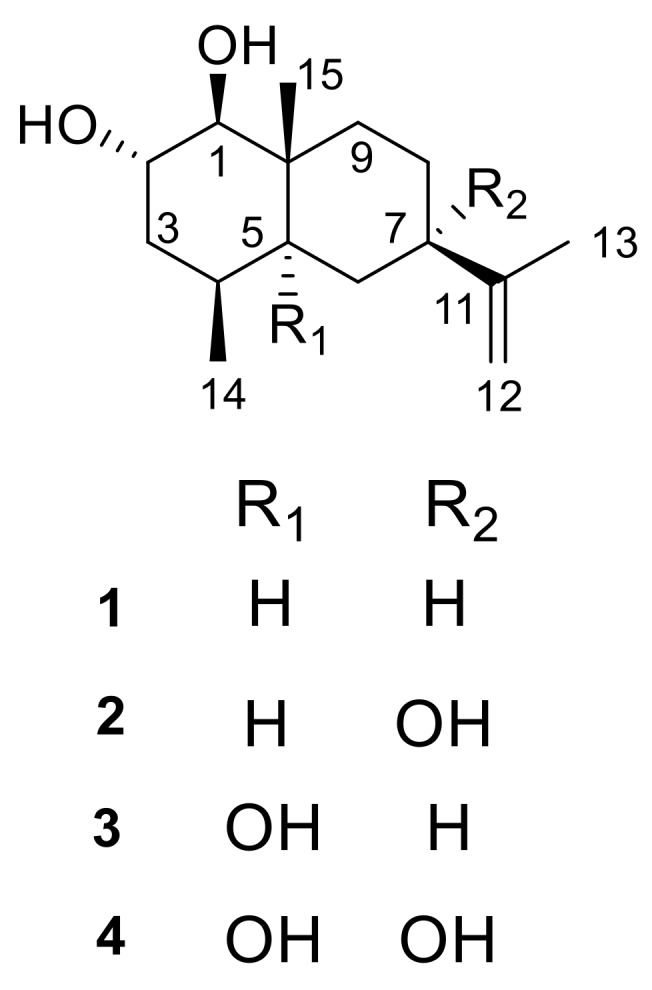
Chemical structures of compounds **1**–**4** from *Penicillium* sp. J-54.

Mangrove forests, the unique forest ecosystems distributed in most tropical and subtropical regions, are an important resource of endophytic fungi that have been proved to be an important source of structurally and biologically diverse substances [1,2,3,4,5,6,7,8,9] such as peniphenones A–D, aniquinazolines A–D, phomazines A–C, and so on [10,11,12]. In order to pursue bioactive products from mangrove fungus, the secondary metabolites of mangrove endophytic fungus *Penicillium* sp. FJ-1 isolated from the stem of *Ceriops tagal* were studied, and a new drimane-type sesquiterpene [13] with antibacterial activity has been reported in our previous research. In our continuous research, four eudesmane-type new sesquiterpenoids, penicieudesmol A–D (**1**–**4**) (Figure 1), were obtained from the culture broth of the *Penicillium* sp. J-54 isolated from the healthy leaves of *Ceriops tagal* collected in Dong Zhai Gang Mangrove Reserve in Hainan. Herein, we described the isolation, structure determination, and biological activities of the new sesquiterpenoids **1**–**4**.

## 2. Results

### 2.1. Structural Elucidation

Penicieudesmol A (**1**), a white powder, had the molecular formula of C_15_H_26_O_2_ determined by HREIMS at *m*/*z* 238.1931 [M]^+^ (calcd. for C_15_H_26_O_2_, *m*/*z* 238.1933). The ^1^H-NMR spectrum of **1** clearly exhibited two olefinic protons (*δ*_H_ 4.67, 4.64), three methyl groups (*δ*_H_ 1.68, 0.85, 0.81), and five methine protons (*δ*_H_ 3.54, 2.74, 1.71, 1.90, 1.31). The ^13^C NMR spectrum combined with the DEPT spectrum (Figure S2) implied a total of 15 carbon resonances including three methyl carbons (*δ*_C_ 21.1, 16.0, 15.6), five methylene carbons (including one sp^2^ methylene carbon and four sp^3^ methylene carbons), five methine carbons (including two oxygen bearing methine carbons and three sp^3^ methine carbons), and two quaternary carbons (*δ*_C_ 150.3, 39.2). The 1D-NMR data of **1** (Table 1) combined with the sequential ^1^H-^1^H COSY correlations of H-1/H-2/H-3/H-4/H-5/H-6/ H-7/H-8/H-9, as well as the key HMBC from H_3_-14 to C-3/C-4/C-5, H_3_-15 to C-1/C-9/C-5, and H_3_-13 to C-7/C-11/C-12, suggested an eudesmane-type skeleton for **1**. By comparison, above data (Table 1) were very close to that of the known compound nardoeudesmol A [14] with the eudesmane-type skeleton. The major difference between them pointed to the additional of a methine (*δ*_C_ 33.7, C-4) and a methyl (*δ*_C_ 15.6, C-14), as well as the absence of two olefinic carbon (*δ*_C_ 146.4, C-4 and *δ*_C_ 109.7, C-14) in **1** based on the key HMBC from H_3_-14 to C-3/C-4/C-5. The relative configuration of **1** was identical to the ROESY experiment (Figure 3), such that the observed cross-correlation peaks from H_3_-15 and H_3_-14 to H-2, as well as from H-1 and H-7 to H-5, proved H_3_-15, H_3_-14, and H-2 were on the same side of the molecular plane and H-1, H-5, and H-7 were on the same side. The large coupling constants (9.2 Hz) between H-1 and H-2 characterised the *trans*-diaxial relationship. Moreover, the absolute configuration of the 1,2-diol moiety in **1** was determined by the in situ dimolybdenum CD method developed by Snatzke and Frelek [14,15,16]. On the basis of the empirical rule proposed by Snatzke, the positive Cotton effect observed at around 310 and 400 nm, respectively, in the induced CD spectrum (Figure 4a) permitted one to assign the 1*S* and 2*S* absolute configuration. Therefore, the absolute configuration of penicieudesmol A was deduced to be 1*S*, 2*S*, 4*S*, 5*S*, 7*R,* and 10*R*.

Penicieudesmol B (**2**) was isolated as a white powder with a molecular formula C_15_H_26_O_3_ determined by its HREIMS at *m*/*z* 254.1878 [M]^+^ (calcd. for *m*/*z* 254.1882). The similarity of 1D and 2D NMR data between **2** (Table 1) and **1** indicated their similar planar structure. The only difference between these two compounds was that H-7 in **1** was substituted by a hydroxyl in **2**, which was proved by the obvious downfield shift of C-7 (*δ*_C_ 72.7) and the HMBC correlations from 7-OH to C-7/C-8 and H_3_-13 to C-7, together with the HREIMS. The relative configuration of **2** was identical with that of **1** by the large coupling constants (9.1 Hz) between H-1 and H-2, as well as the ROESY correlations (Figure 3). In addition, the 2*S* configuration of compound **2** was clearly defined by the observed chemical shift differences Δ*δ_S_*_−*R*_ by the modified Mosher’s method (Figure 4b) [12]. So, the stereogenic centers of penicieudesmol B were determined as 1*S*, 2*S*, 4*S*, 5*S*, 7*S,* and 10*R*.

Penicieudesmol C (**3**) was obtained as yellow oil with the molecular formula C_15_H_26_O_3_ determined according to the HREIMS peak at *m*/*z* 254.1880 [M]^+^ (calcd. for *m*/*z* 254.1882), indicating an isomer of **2**. The ^1^H and ^13^C NMR data of **3** (Table 2) showed high similarity to those of **2**, except for the location of hydroxyl in the two compounds. The sequential ^1^H-^1^H COSY correlations of H-6/H-7/H-8/H-9, together with the key HMBC correlations from 5-OH, H_3_-14, and H_3_-15, as well as H-7 to C-5, from H_3_-15 to C-9, and from H_3_-13 to C-7 displayed that 7-OH in **2** shifted to 5-OH in **3**. The relative and absolute configuration of **3** was determined to be consistent with that of **2** through the same method (Figures 3 and 4b). Hence, the stereogenic centers of penicieudesmol C were determined as 1*S*, *2*S, 4*S*, 5*R*, 7*R,* and 10*S*.

**Figure 2:**
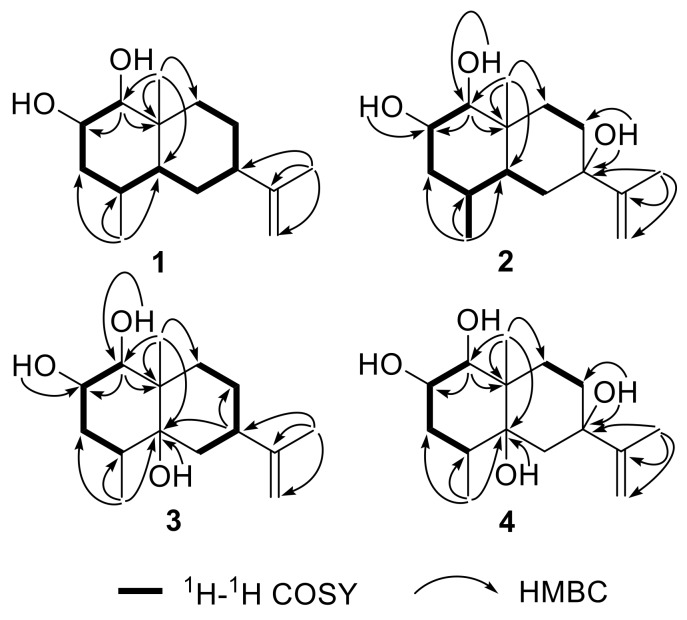
The key 2D-NMR correlations for compounds **1**−**4**.

**Figure 3:**
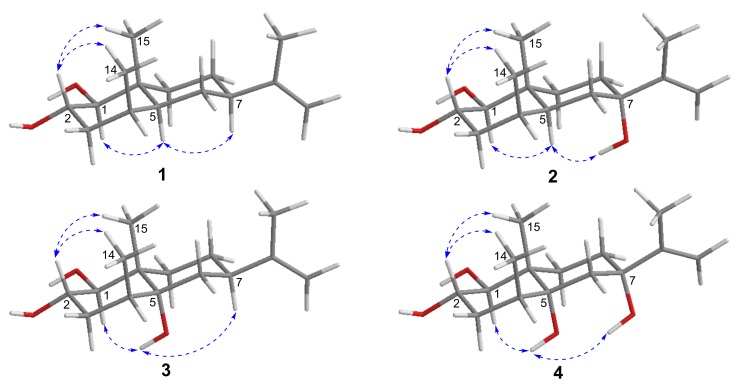
Key ^1^H–^1^H REOSY correlations of compounds **1**–**4**.

**Figure 4:**
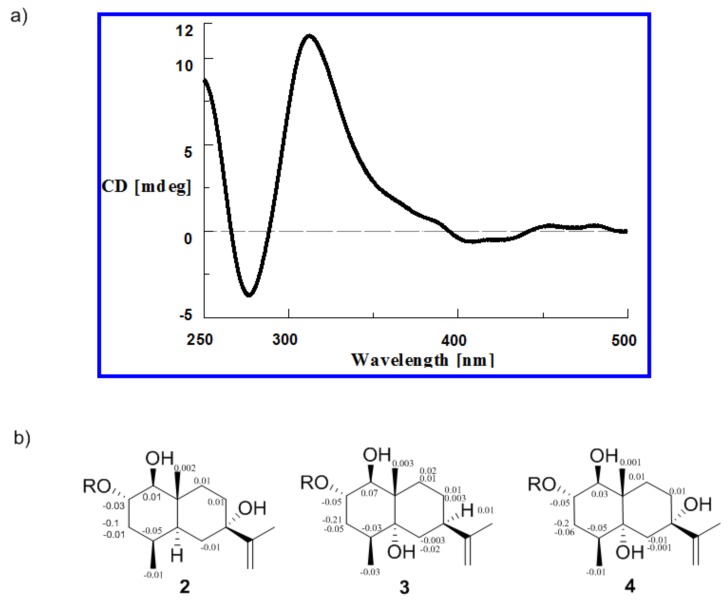
(**a**) CD spectrum of **1** in DMSO containing Mo_2_(OAc)_4_ with the inherent CD spectrum; (**b**) Δ*δ* (=*δS* − *δR*) values for (*S*)- and (*R*)-MTPA esters of **2**–**4**.

Penicieudesmol D (**4**) was also obtained as yellow oil. The HREIMS displayed a quasi-molecular ion peak at *m*/*z* 270.1833 [M]^+^ (calcd. for *m*/*z* 270.1831), indicating the molecular formula C_15_H_26_O_4_. The ^1^H and ^13^C NMR data of compound **4** was very close to those of compound **3**. According to the HREIMS of them, hydrogen atoms in **3** were substituted by a hydroxy group in **4**. The sequentia ^1^H–^1^H COSY correlations of H-1/H-2/H-3/H-4 and H-8/H-9 combined the key HMBC correlations (Figure 2) from 7-OH to C-7 and C-8, as well as H_3_-13 to C-7, along with the downfiled shifts and ^13^C multiplicity of C-7 (*δ*_C_ 75.1). Table 2 suggests that the substituent hydrogen atoms were H-7 in **4**. The relative and absolute configuration of **4** was determined to be consistent with that of **2** and **3** via the same method (Figure 3 and Figure 4b). Consequently, the stereogenic centers of penicieudesmol D were determined to be 1*S*, 2*S,* 4*S*, 5*R*, 7*S,* and 10*S*. 

### 2.2. The Bioactivities of Compounds ***1**–**4*** from Penicillium sp. J-54

All the compounds (**1**–**4**) were evaluated for their cytotoxic activity against K-562, SEL-7420, and SGC-7721 cell lines using the MTT method in vitro [17] and antimicrobial activity against *Candida albicans* and *Staphylococcus aureus* using the filter paper disc agar diffusion method [18]. The results showed that compound **2** exhibited weak cytotoxicity against K-562 with IC_50_ value of 90.1 µM, with paclitaxel as the positive control (IC_50_ = 9.5 µM). Unfortunately, none of these compounds showed antimicrobial activity.

## 3. Materials and Methods

### 3.1. General Experimental Procedures

Silica gel (60–80, 200–300 mesh, Qingdao Marine Chemical Co., Ltd., Qingdao, China), ODS gel (20–45 µm, Fuji Silysia Chemical Co., Ltd., Greenville, NC, USA), and Sephadex LH-20 (Merck, Kenilworth, NJ, USA) were used for column chromatography. TLC was conducted on precoated silica gel G plates (Qingdao Marine Chemical Co., Ltd.), and spots were detected by spraying with 5% H_2_SO_4_ in EtOH followed by heating. Optical rotation was measured on a Rudolph Autopol III polarimeter. UV spectra were performed on a Shimadzu UV-2550 spectrometer (Beckman, Brea, CA, USA). IR absorptions were obtained on a Nicolet 380 FT-IR instrument (Thermo, Waltham, MA, USA) using KBr pellets. 1D and 2D-NMR spectra were recorded on Bruker AV III spectrometer (Bruker, Billerica, MA, USA) (^1^H-NMR at 500 MHz and ^13^C NMR at 125 MHz) using TMS as the internal standard. Chem3D Pro 14.0 (PerkinElmer, Waltham, MA, USA) was used for building these 3D models and calculating energy minimizations.

### 3.2. Fungal Material

*Penicillium* sp. J-54 was isolated from the healthy leaves of *Ceriops tagal*, which were collected in Dong Zhai Gang Mangrove Reserve in Hainan province, in July 2011. The endophytic fungus was identified based on the DNA sequences of 18S rDNA gene. For identification of its 18S rDNA gene sequences, the *Penicillium* sp. J-54 was cultured in potato dextrose agar for five days. The mycelium was ground to a fine powder in liquid N_2_, then genomic DNA was extracted, and 18S rDNA region was amplified by PCR using primers NS1 (5′-GTAG TCATATGCTTGTCTC-3′) and NS6 (5′-GCATCACAGACCTGTTATTGCCTC-3′). PCR products were sequenced (Applid Biosystems 3730 XL Genetic Analyzer, Applied Biosystems Inc., Foster City, CA, USA). The producing strain was prepared on PDA medium and stored in our Lab. at 4 °C.

### 3.3. Fermentation and Extraction 

*Penicillium* sp. J-54 was cultured in PDB (the potato liquid media consisting of 200.0 g/L potato, 20.0 g/L glucose, and 1000 mL deionized water) at 29 °C and 130 rpm for 72 h. 20 mL of the seed culture was inoculated into each 1000 mL Erlenmeyer flask of production medium composed of (per litre) 20.0 g potato, 0.4 g glucose, and 400 mL deionized water; the pH was adjusted 7.0. They were cultivated in static for 4 weeks after being incubated at 29 °C for 7 days on a rotary shaker at 130 rpm. The liquid filtrate from 100 L of fermentation broth was collected and extracted four times with ethyl acetate (1000 mL × 4 times) at room temperature.

### 3.4. Purification and Identification

The obtained EtOAc crude extract (35.5 g), which was separated into 10 fractions (Fr.1–Fr.10) on silica gel (100.0 g, 200–300 mesh) column chromatography (CC) (4 × 60 cm), eluted with a gradient elution of CHCl_3_-MeOH (*v*/*v*, 1:0 to 0:1, each 1000 mL). Fr.2 (3.3 g) was purified by ODS column chromatography (CC) (2.5 × 40 cm) with gradient of Water-MeOH (*v*/*v*, 30:70, 40:60, 50:50, 60:40, 70:30, 80:20, 90:10, 100:0, each 1 L) to get five subfractions (Fr.2.1–Fr.2.5). Fr.2.4 was submitted to Sephadex LH-20 (2 × 30 cm), eluted with MeOH (500 mL), then further separated on a silica gel CC (1 × 20 cm) eluted with CHCl_3_-MeOH step gradient (*v*/*v*, 200:1 to 20:1) to yield compound **1** (3.8 mg) and compound **3** (4.1 mg). Fr.3 (2.2 g) was separated on a silica gel CC (2.5 × 40 cm) eluted with CHCl_3_-MeOH step gradient (*v*/*v*, 1:0 to 10:1) to yield eleven subfractions (Fr.3-1–Fr.3-11). Fr.3-4 (451.5 mg) was applied to Sephadex LH-20 (2 × 30 cm) with CHCl_3_-MeOH (*v*/*v*, 1:1, 400 mL) as eluent, and then further purified again by silica gel CC (1 × 20 cm) with eluting of CHCl3-MeOH (*v*/*v*, 50:1, 1500 mL) to obtain compound **4** (3.1 mg). Fr.4 (2.5 g) was separated on a silica gel CC (2.5 × 40 cm) eluted with CHCl_3_-MeOH step gradient (*v*/*v*, 1:0 to 0:1) to yield eight subfractions. (Fr.4-1–Fr.4-8). Fr.4-5 (3.7 g) yielded compound **2** (3.5 mg) after purified by silica CC (1 × 20 cm) eluted with CHCl_3_-MeOH (*v*/*v*, 80:1, 1.5 L).

Compound **1**: white power; [α]^20^_D_ + 8.0 (c = 0.5, MeOH); IR (KBr) ν_max_: 3417.4, 2930.9, 1643.2, 1384.2, 1025.4, 438.9 cm^−1^; HREIMS: *m*/*z* 238.1931 [M]^+^ (calcd. for C_15_H_26_O_2_, 238.1933); ^1^H and ^13^C-NMR data: see Table 1.

Compound **2**: white power; [α]^20^_D_ + 80.0 (c = 0.5, MeOH); IR (KBr) ν_max_: 3424.5, 2925.7, 1655.4, 1023.4, 582.7 cm^−1^; HREIMS: *m*/*z* 254.1878 [M]^+^ (calcd. for C_15_H_26_O_3_, 254.1882); ^1^H and ^13^C-NMR data: see Table 1.

Compound **3**: yell ow oil; [α]^20^_D_ + 31.0 (c = 0.5, MeOH); IR (KBr) ν_max_: 3423.2,2923.9,1636.7, 1384.4, 1044.5, 668.2 cm^−1^; HREIMS: *m*/*z* 254.1880 [M]^+^ (calcd. for C_15_H_26_O_3_, 254.1882); ^1^H and^13^C-NMR data: see Table 2.

Compound **4**: yellow oil; [α]^20^_D_ + 12.0 (c = 0.5, MeOH); IR (KBr) ν_max_: 3415.8, 2923.9, 1636.7, 1384.1, 1029.5, 462.3 cm^−1^; HREIMS: *m*/*z* 270.1833 [M]^+^ (calcd. for C_15_H_26_O_4_, 270.1831); ^1^H and ^13^C-NMR data: see Table 2.

### 3.5. Preparation of S-MTPA and R-MTPA Esters ***1a***, ***1b***, ***2a***, ***2b***, ***3a***, and ***3b*** of Compounds ***1***, ***2***, and ***3***

Compound **2** (1 mg) was dissolved in 1 mL CH_2_Cl_2_, and 4-dimethylaminopyridine (3 mg) and (*R*)-MTPACl (10 μL) were added. The reaction was stirred for 5 h at room temperature. Then, 1 mL of H_2_O was added to stop the reaction and to extract the solution three times with CH_2_Cl_2_ (5 mL each). Finally, the residue was purified by semipreparative HPLC (80% MeOH-H_2_O) after removal of CH_2_Cl_2_ under reduced pressure to obtain (*S*)-MTPA ester **2a** (1 mg, *t_R_* = 7.84 min). By the same procedure, (*R*)-MTPA ester **2b** (1 mg, *t_R_* = 8.17 min), (*S*)-MTPA ester **3a** (1 mg, *t_R_* = 8.55 min), (*R*)-MTPA ester **3b** (1 mg, *t_R_* = 8.64 min), (*S*)-MTPA ester **4a** (1 mg, *t_R_* = 6.79 min), and (*R*)-MTPA ester **4b** (1 mg, *t_R_* = 6.98 min) were got via the reaction of **2**, **3**, and **4** (1 mg, each) with (*S*)-MTPACl, (*R*)-MTPACl, (*S*)-MTPACl, (*R*)-MTPACl, and (*S*)-MTPACl, respectively [12].

### 3.6. Absolute Configuration of the *1, 2*-Diol Moiety in ***1***

A mixture of diol-Mo_2_(OAc)_4_ (1:1.3) for **1** was subjected to CD measurements at a concentration of 0.5 mg/mL in HPLC grade DMSO dried with 4 Å molecular sieves, according the literature report [19]. The first CD spectrum was recorded after mixing immediately, and the CD spectrum was recorded again after mixing for 10 min. The inherent CD was subtracted. The observed signs of the diagnostic bands at about 310 and 400 nm in the induced CD spectrum were correlated to the absolute configuration of the 1, 2-diol moiety.

### 3.7. Bioassays

The cytotoxic activity for compounds **1**–**4** were tested against three cell lines including human hepatic carcinoma cell lines (SEL-7420), gastric cell lines (SGC-7721), and leukemia cell lines (K-562). These cell lines were purchased from Shang Hai Cell Bank of Chinese Academy of Sciences. The purity of the tested compounds and paclitaxel (PTX) was determined to be over 95% using the chromatography. The cytotoxic effects on these tests cell were assessed by the IC_50_ values and determined by the MTT [3-(4,5-dimethylthiazol-2-yl)-2,5-diphenyl-tetrazolium bromide] colometric method as described in reference [17]. Each set of tests was conducted three times to confirm reproducibility of the results. These compounds were dissolved in DMSO, PTX was used as a positive control, and the medium without test compound was used as a negative control in the bioassay.

The antimicrobial activity of compounds **1**–**4** against *C. albicans* and *S. aureus* were also evaluated using the 2-fold dilution method [18]. The tested strains were cultivated in YPD broth for *C. albicans* and LB broth for bacteria at 28 °C. The test compounds were dissolved in DMSO at different concentrations from DMSO at different concentrations from 1000 to 7.8 μg/mL (from 6.25 to 0.025 μg/mL for the positive controls) by the continuous 2-fold dilution methods in 96-well plates. Each well contains 100 μL of contents composed of 20 μL of inoculums (5 × 10^5^ CFU/mL), test compounds, and YPD or LB media. The microtiter plates were incubated at 28 °C for 24 h and were examined for microbes’ growth by turbidity in daylight. Chlorhexidine acetate and kanamycin sulfate were used as positive controls for *C. albicans* and *S. aureus*, respectively.

## 4. Conclusions

Four new eudesmane-type sequiterpenes (**1**–**4**) were isolated from the PDB fermentation broth of the mangrove-derived endophytic fungus *Penicillium* sp. J-54 originated from the healthy leaves of *Ceriops tagal* collected in Dong Zhai Gang Mangrove Reserve in Hainan. Their structures were determined by spectroscopic methods, the in situ dimolybdenum CD method, and the modified Mosher’s method. Compound **2** exhibited weak cytotoxicity against K-562 with an IC_50_ value of 90.1 μM. The results proved that mangrove endophytic fungi are the source of new bioactive substances.

## Figures and Tables

**Table 1:** ^1^H and ^13^C-NMR Data for **1** and **2** (500 and 125 MHz, DMSO-*d*_6_, *δ* in ppm).

Position	1		2	
	*δ*_C_, Type	*δ*_H_ mult. (*J* in Hz)	*δ*_C_, Type	*δ*_H_, mult. (*J* in Hz)
1	83.9, CH	2.74, dd, (9.2, 4.0)	83.8, CH	2.77, dd, (9.2, 3.9)
2	66.7, CH	3.54, m	66.6, CH	3.64, m
3	40.5, CH_2_	1.79, m	40.5, CH_2_	1.68, m
		1.43, m		1.42, m
4	33.7, CH	1.71, m	33.3, CH	1.59, m
5	45.7, CH	1.31, m	39.1, CH	1.76, m
6	31.3, CH_2_	1.41, m	35.8, CH_2_	1.51, m
		1.25, m		1.11, d, (13.0, 2.5)
7	45.6, CH	1.90, m	72.7, qC	
8	26.2, CH_2_	1.44, m	30.4, CH_2_	1.54, m
		1.27, m		1.33, m
9	40.3, CH_2_	1.67, m	35.9, CH_2_	1.66, m
		0.98, m		1.35, m
10	39.2, qC		38.9, qC	
11	150.3, qC		153.2, qC	
12	108.7, CH_2_	4.67, s	108.5, CH_2_	4.96, d, (1.8)
		4.64, s		4.68 d, (1.8)
13	21.1, CH_3_	1.68, s	19.2, CH_3_	1.74, s
14	15.6, CH_3_	0.81, s	14.6, CH_3_	0.79, s
15	16.0, CH_3_	0.85, d, (7.6)	15.9, CH_3_	0.86, d, (7.6)
1-OH				4.38, d, (3.7)
2-OH		4.41, d, overlap		4.37, d, (3.7)
5-OH		4.41, d, overlap		
7-OH				4.22, s

**Table 2:** ^1^H and ^13^C NMR Data for **3** and **4** (500 and 125 MHz, DMSO-*d*_6_, *δ* in ppm).

Position	3		4	
	*δ*_C_, Type	*δ*_H_, mult. (*J* in Hz)	*δ*_C_, Type	*δ*_H_ mult. (*J* in Hz)
1	77.8, CH	3.45, dd, (9.1, 2.5)	77.5, CH	3.40, d, (9.2)
2	67.7, CH	3.66, m	67.3, CH	3.60, m
3	35.9, CH_2_	2.06, m	35.0, CH_2_	1.93, m
		1.48, m		1.40, m
4	41.2, CH	1.71, m	40.5, CH	1.67, m
5	75.3, qC		76.7, qC	
6	37.1, CH_2_	1.79, m	38.7, CH_2_	1.99, d, (14.0)
		1.25, dd, (13.3, 3.0)		1.15, d, (14.0)
7	39.2, CH	2.53, m	75.1, qC	
8	25.4, CH_2_	1.49, m	30.3, CH_2_	1.46, m
		1.34, m		
9	33.3, CH_2_	1.76, m	29.8, CH_2_	1.76, m
		1.43, m		1.40, m
10	41.9, qC		42.1, qC	
11	150.7, qC		151.6, qC	
12	108.7, CH_2_	4.73, d, (1.6)	109.3, CH_2_	4.96, s
		4.75, d, (1.6)		4.74, s
13	21.3, CH_3_	1.67, s	19.0, CH_3_	1.74, s
14	17.8, CH_3_	0.96, d, (7.8)	17.4, CH_3_	0.95, d, (7.8)
15	16.9, CH_3_	0.86, s	16.7, CH_3_	0.87, s
1-OH		4.29, d, (3.7)		4.26, br s
2-OH		4.37, d, (2.8)		4.20, br s
5-OH		3.74, s		5.66, s
7-OH				5.63 s

## Supplementary Materials

The NMR and HREIMS spectra for **1**–**4** and the ^1^H-NMR spectra for *S*-MTPA and *R*-MTPA esters are available online at http://www.mdpi.com/1660-3397/16/4/108/s1.
