# Supplementary material for: New Eudesmane-Type Sesquiterpenoids from the Mangrove-Derived Endophytic Fungus Penicillium sp. J-54
Source: Mar Drugs. 2018 Mar 28;16(4):108. doi: 10.3390/md16040108 (PMC5923395; doi:10.3390/md16040108)

Supplementary Information

Table of Contents

The 18S rRNA gene sequences data of *Penicillium* sp. J-54

Figure S1. The ^{1}H NMR (500 MHz, DMSO-d_6) spectrum of compound **1**

Figure S2. The ^{13}C NMR and DEPT135 (125 MHz, DMSO-d_6) spectrum of compound **1**

Figure S3. The ^{1}H-^{1}H COSY (500 MHz, DMSO-d_6) spectrum of compound **1**

Figure S4. The HSQC (500 MHz, DMSO-d_6) spectrum of compound **1**

Figure S5. The HMBC (500 MHz, DMSO-d_6) spectrum of compound **1**

Figure S6. The ROESY (500 MHz, DMSO-d_6) spectrum of compound **1**

Figure S7. The HRESIMS spectrum of compound **1**

Figure S8. The ^{1}H NMR (500 MHz, DMSO-d_6) spectrum of compound **2**

Figure S9. The ^{13}C NMR and DEPT135 (125 MHz, DMSO-d_6) spectrum of compound **2**

Figure S10. The ^{1}H-1H COSY (600 MHz, DMSO-d_6) spectrum of compound **2**

Figure S11 The HSQC (500 MHz, DMSO-d_6) spectrum of compound **2**

Figure S12. The HMBC (500 MHz, DMSO-d_6) spectrum of compound **2**

Figure S13. The ROESY (500 MHz, DMSO-d_6) spectrum of compound **2**

Figure S14. The HRESIMS spectrum of compound **2**

Figure S15. The ^{1}H NMR (500 MHz, DMSO-d_6) spectrum of compound **3**

Figure S16. The ^{13}C NMR and DEPT135 (125 MHz, DMSO-d_6) spectrum of compound **3**

Figure S17. The ^{1}H-^{1}H COSY (500 MHz, DMSO-d_6) spectrum of compound **3**

Figure S18. The HSQC (500 MHz, DMSO-d_6) spectrum of compound **3**

Figure S19. The HMBC (500 MHz, DMSO-d_6) spectrum of compound **3**

Figure S20. The ROESY (500 MHz, DMSO-d_6) spectrum of compound **3**

Figure S21. The HRESIMS spectrum of compound **3**

Figure S22. The ^{1}H NMR (500 MHz, DMSO-d_6) spectrum of compound **4**

Figure S23. The ^{13}C NMR and DEPT135 (125 MHz, DMSO-d_6) spectrum of

compound **4**

Figure S24. The ^{1}H-^{1}H COSY (500 MHz, DMSO-d_6) spectrum of compound **4**

Figure S25. The HSQC (500 MHz, DMSO-d_6) spectrum of compound **4**

Figure S26. The HMBC (500 MHz, DMSO-d_6) spectrum of compound **4**

Figure S27. The ROESY (500 MHz, DMSO-d_6) spectrum of compound **4**

Figure S28. The HRESIMS spectrum of compound **4**

Figure S29. The ^{1}H NMR (500 MHz, CD_3OD-d_4) spectrum of *S*-MTPA ester **1a** and *R*-MTPA ester **1b**

Figure S30. The ^{1}H NMR (500 MHz, CD3COCD3-d_6) spectrum of *S*-MTPA ester **2a** and *R*-MTPA ester **2b**

Figure S31. The ^{1}H NMR (500 MHz, CD_3OD-d_4) spectrum of *S*-MTPA ester **3a** and *R*-MTPA ester **3b**

The 18S rDNA Gene Sequences Data of *Penicillium* sp. J-54

5'-GCTCATTAAATCAGTTATCGTTTATTTGATAGTACCTTACTACATGGATACC
TGTGGTAATTCTAGAGCTAATACATGCTACAAACCCCGACTTCAGGAAGGG
GTGTATTTATTAGATAAAAAACCAACGCCCTTCGGGGCTCCTTGGTGAATCA
TAATAACTTAACGAATCGCATGGCCTTGCGCCGGCGATGGTTCATTCAAATT
TCTGCCCTATCAACTTTCGATGGTAGGATAGTGGCCTACCATGGTGGCAACG
GGTAACGGGGAATTAGGGTTCGATTCCGGAGAGGGAGCCTGAGAAACGGC
TACCACATCCAAGGAAGGCAGCAGGCGCGCAAATTACCCAATCCCGATACG
GGGAGGTAGTGACAATAAATACTGATACGGGGCTCTTTCGGGTCTCGTAATT
GGAATGAGAACAATTTAAATCCCTTAACGAGGAACAATTGGAGGGCAAGT
CTGGTGCCAGCAGCCGCGGTAATTCCAGCTCCAATAGCGTATATTAAAGTTG
TTGCAGTTAAAAAGCTCGTAGTTGAACCTTGGGCCTGGCTGGCCGGTCCGC
CTCACCGCGAGTACTGGTCCGGCTGGGCCTTTCCTTCTGGGGAACCTCATG
GCCTTCACTGGCTGTGGGGGGAACCAGGACTTTTACTGTGAAAAAATTAG
AGTGTTCAAAGCAGGCCTTTGCTCGAATACATTAGCATGGAATAATAGAATA
GGACGTGCGGTTCTATTTTGTTGGTTTCTAGGACCGCCGTAATGATTAATAG
GGATAGTCGGGGGCGTCAGTATTCAGCTGTCAGAGGTGAAATTCTTGGATT
TGCTGAAGACTAACTACTGCGAAAGCATTCGCCAAGGATGTTTTCATTAATC
AGGGAACGAAAGTTAGGGGATCGAAGACGATCAGATACCGTCGTAGTCTT
AACCATAAACTATGCCGACTAGGGATCGGACGGGATTCTATGATGACCCGTT
CGGCACCTTACGAGAAATCAAAGTTTTTGGGTTCTGGGGGGAGTATGGTCG
CAAGGCTGAAACTTAAAGAAATTGACGGAAGGGCACCACAAGGCGTGGA
GCCTGCGGCTTAATTTGACTCAACACGGGGAAACTCACCAGGTCCAGACA
AAATAAGGATTGACAGATTGAGAGCTCTTTCTTGATCTTTTGGATGGTGGTG
CATGGCCGTTCTTAGTTGGTGGAGTGATTTGTCTGCTTAATTGCGATAACGA
ACGAGACCTCGGCCCTTAAATAGCCCGGTCCGCATCTGCGGGCCGCTGGC-
3'.

Figure S1. The ^{1}H NMR (500 MHz, DMSO-d_6) spectrum of compound **1**

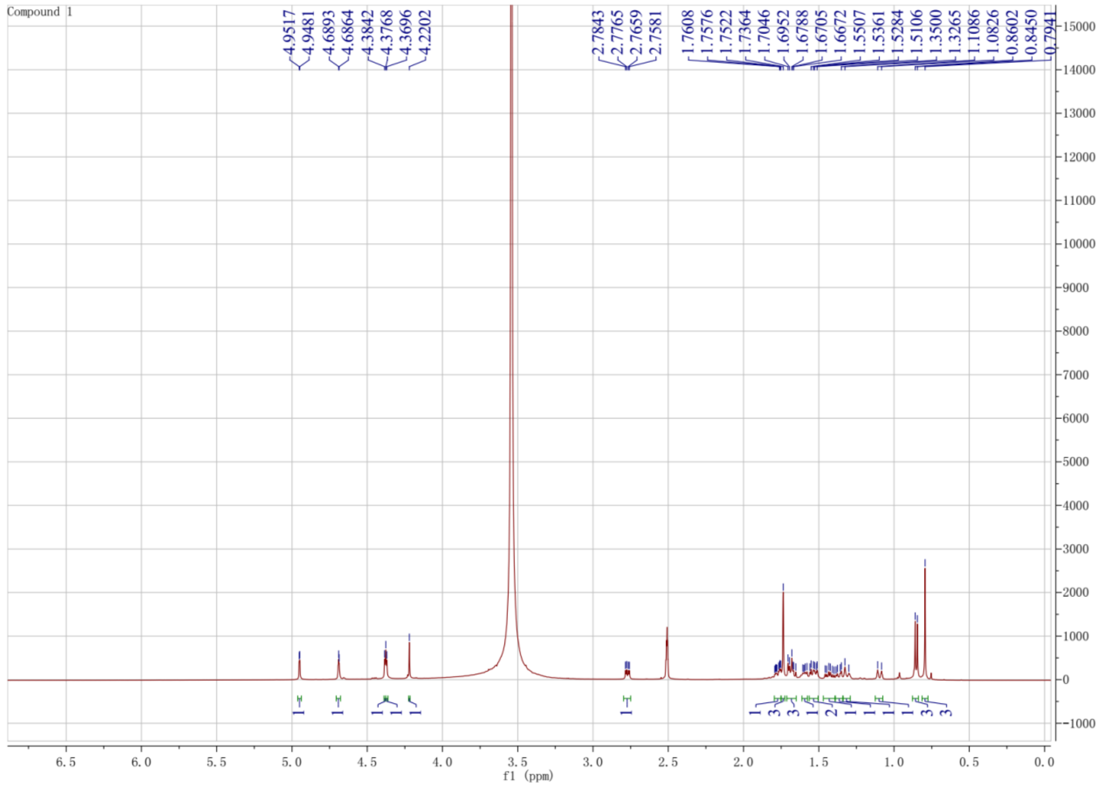

Figure S2. The ^{13}CNMR and DEPT135 (125 MHz, DMSO-d_6) spectrum of compound **1**

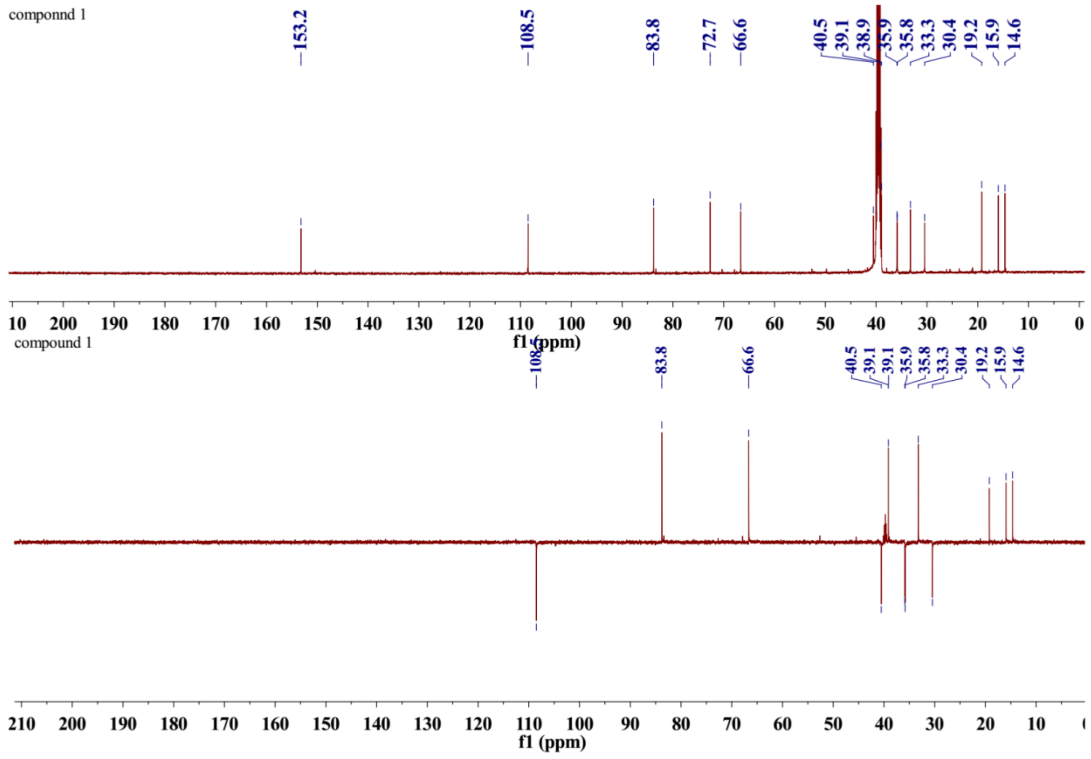

Figure S3. The ^{1}H-^{1}H COSY (500 MHz, DMSO-d_6) spectrum of compound **1**

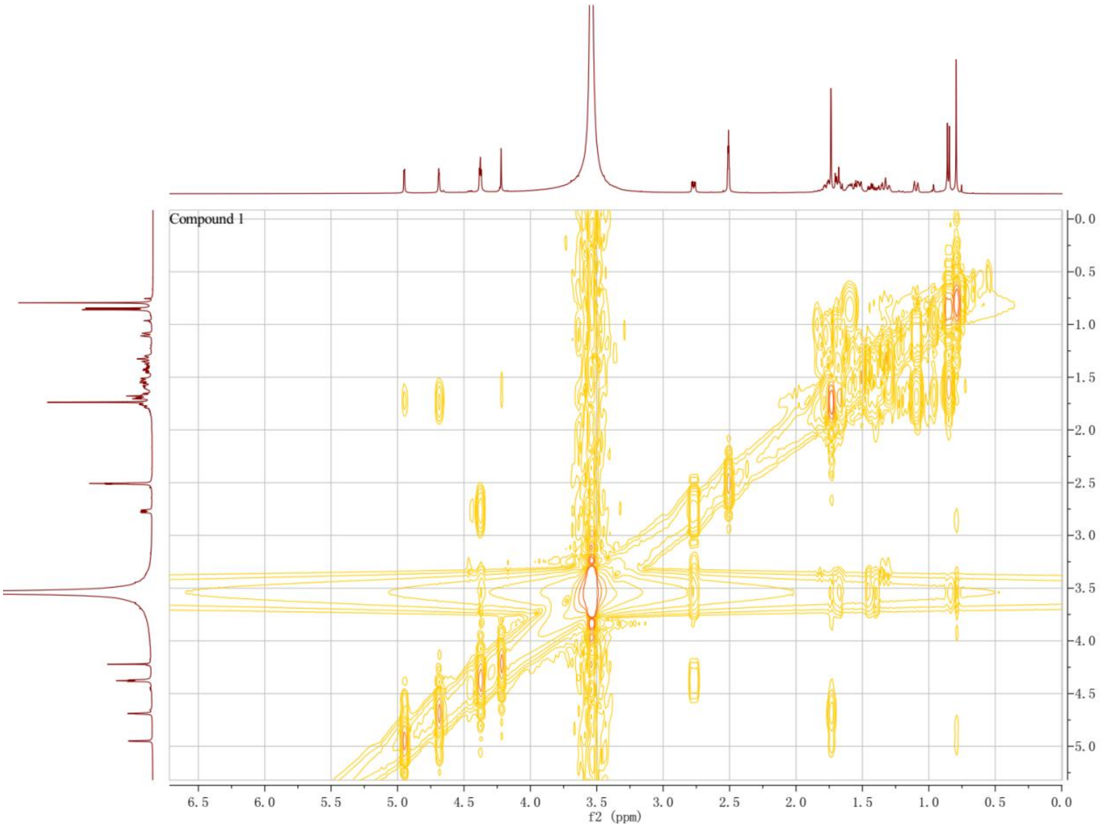

Figure S4. The HSQC (500 MHz, DMSO-d_6) spectrum of compound **1**

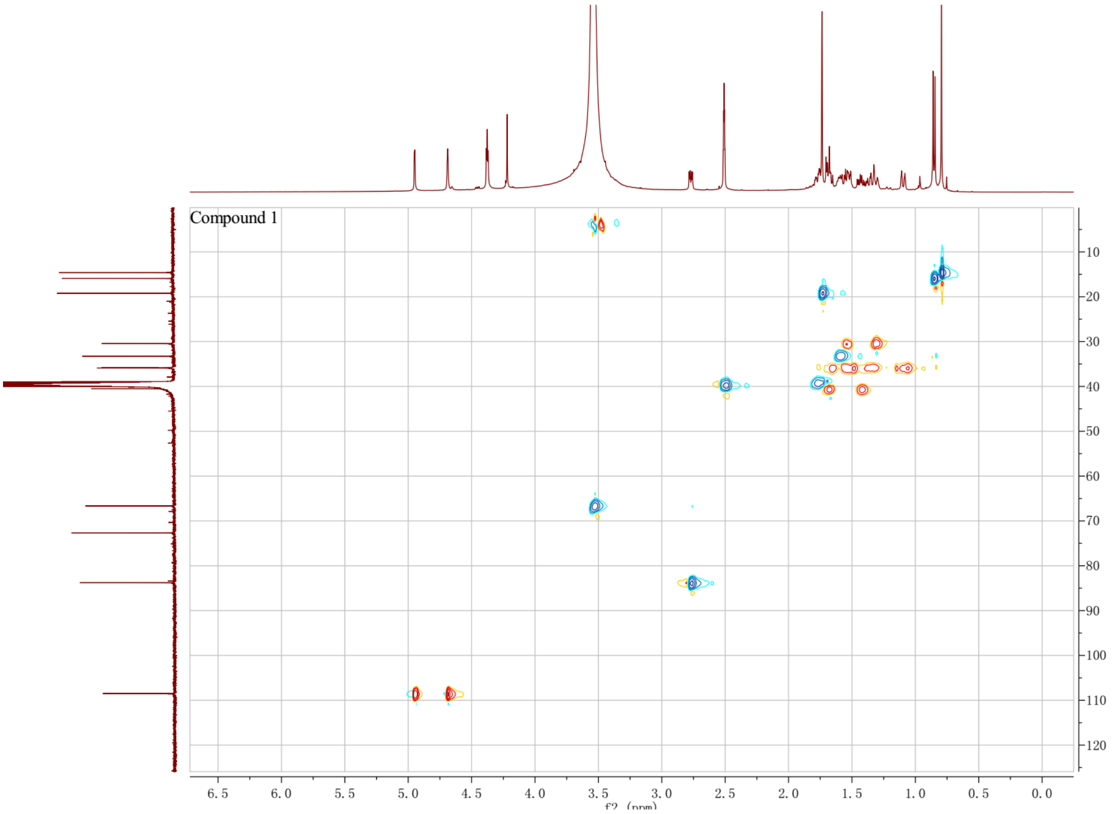

Figure S5. The HMBC (500 MHz, DMSO-d_6) spectrum of compound **1**

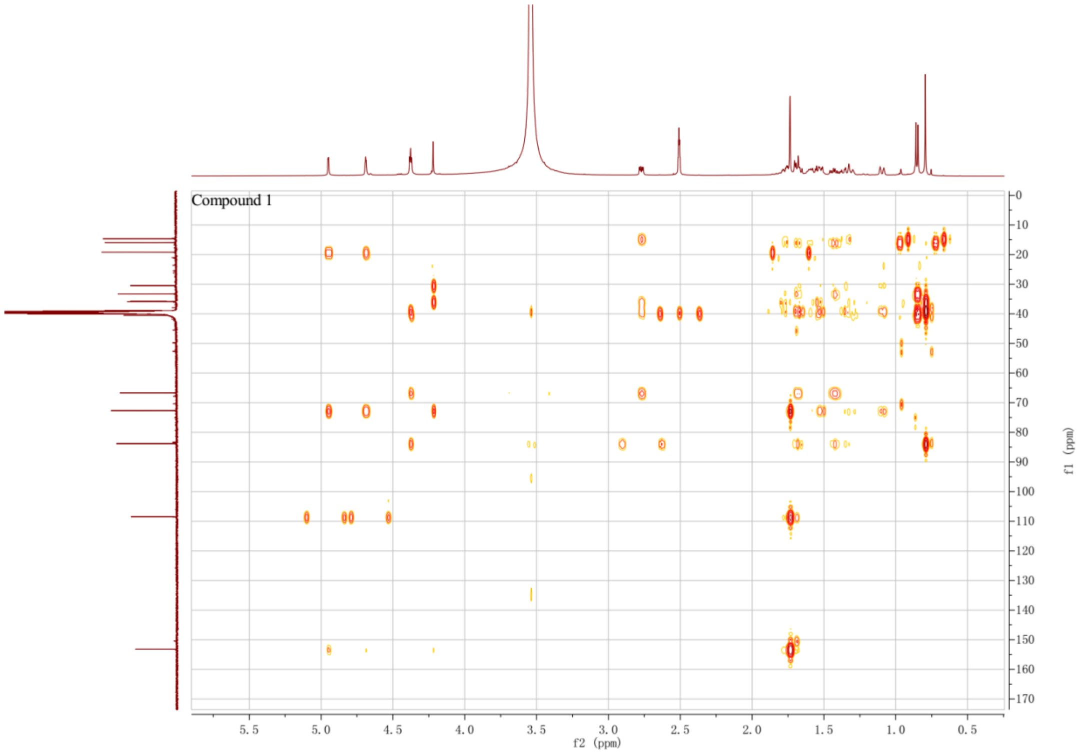

Figure S6. The ROESY (500 MHz, DMSO-d_6) spectrum of compound **1**

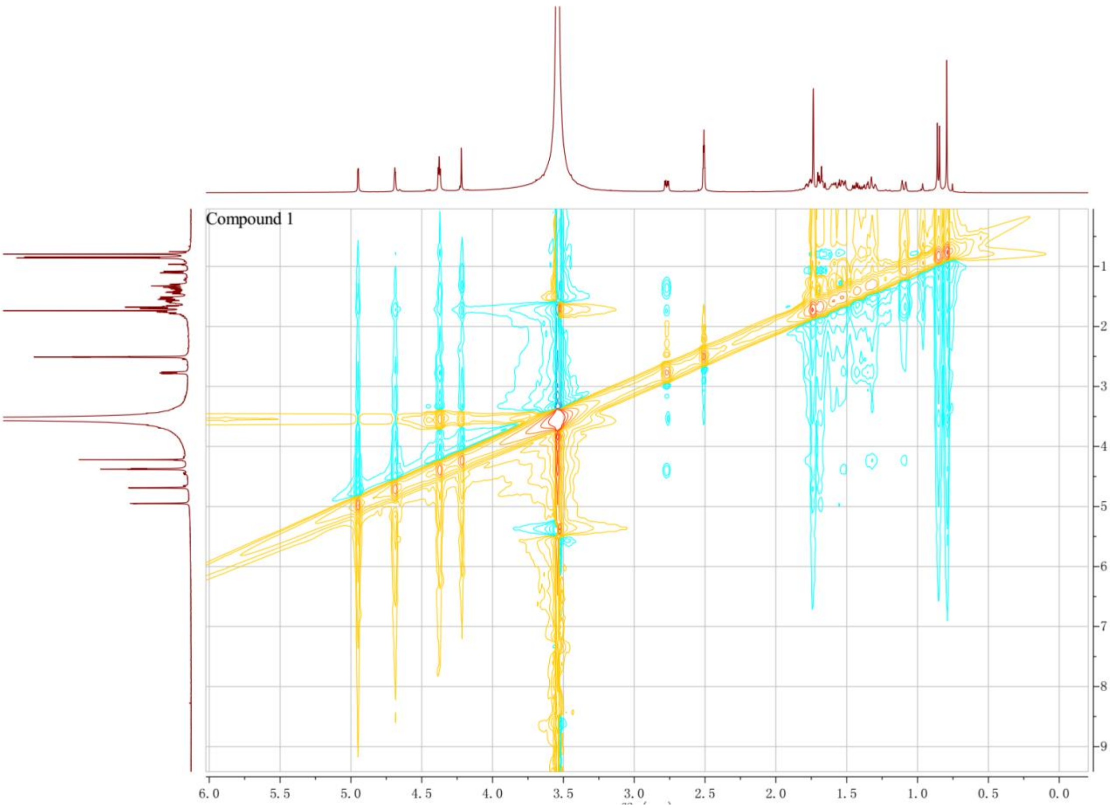

Figure S7. The HRESI spectrum of compound **1**

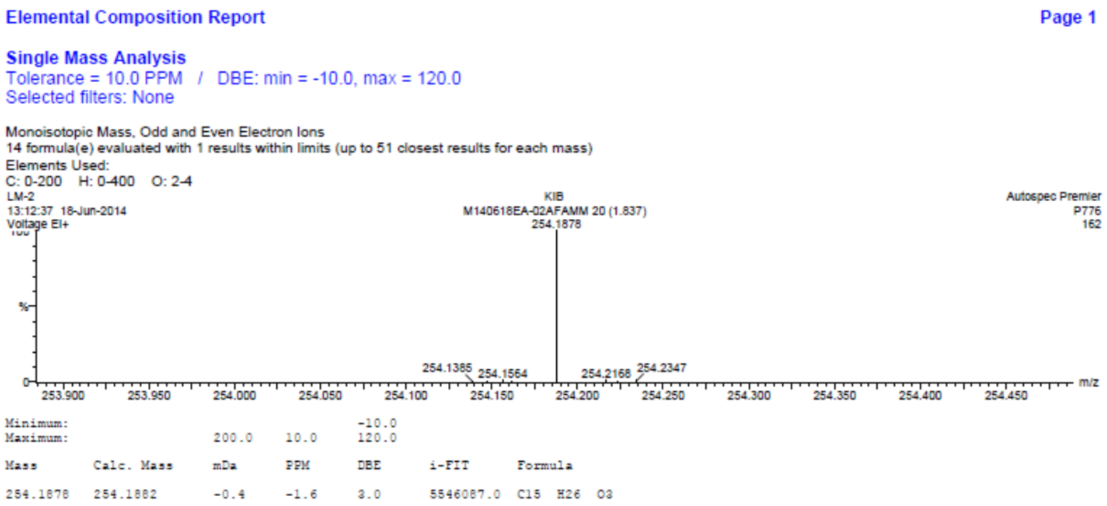

Figure S8. The ^{1}H NMR (500 MHz, DMSO-d_6) spectrum of compound **2**

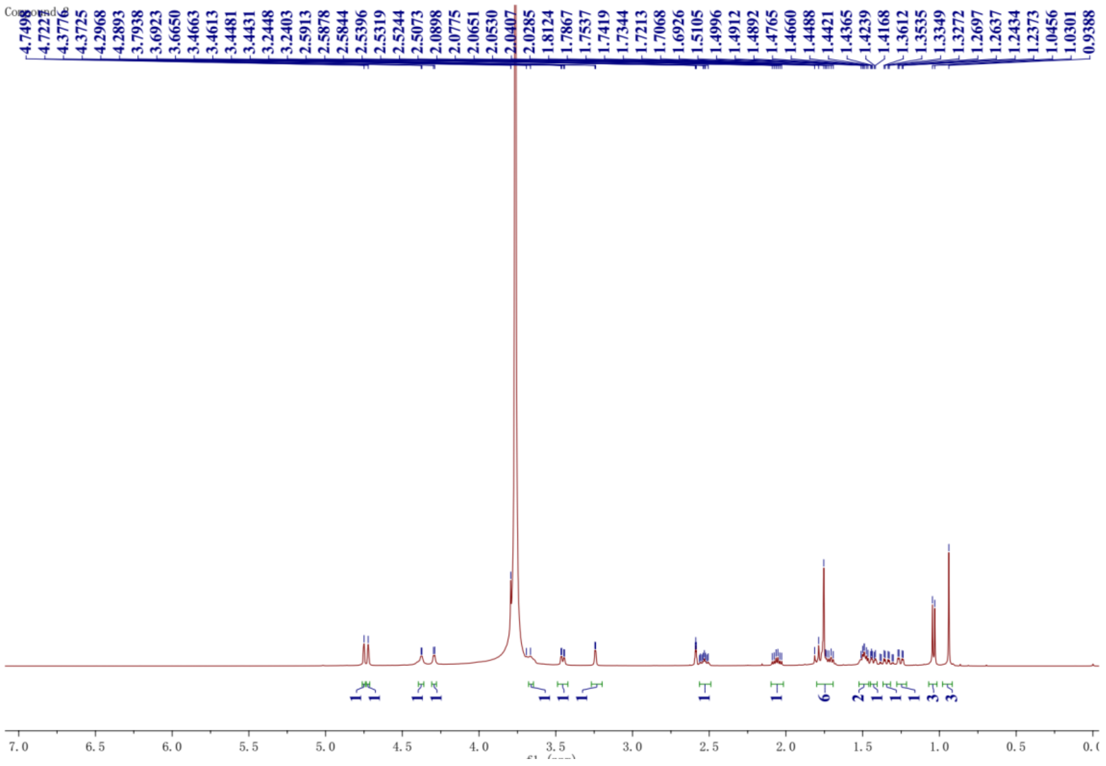

Figure S9. The [13] CNMR and DEPT135 (125 MHz, DMSO-d_6) spectrum of compound **2**

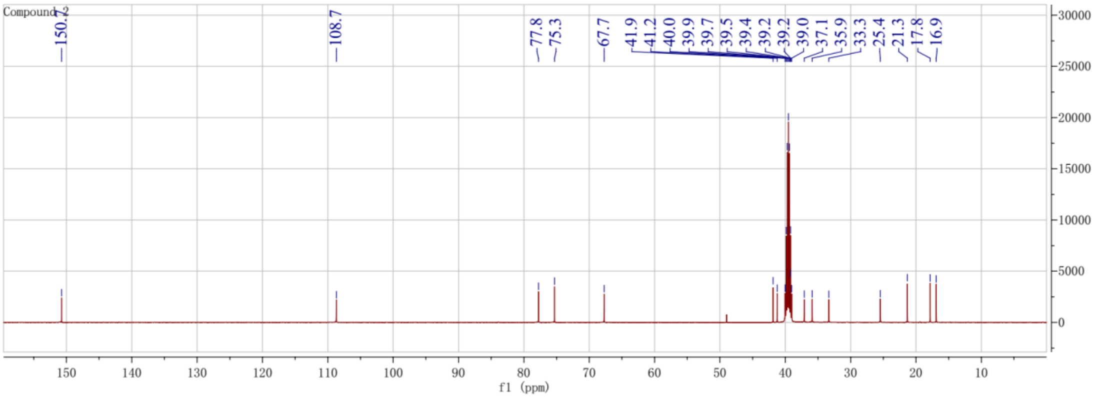

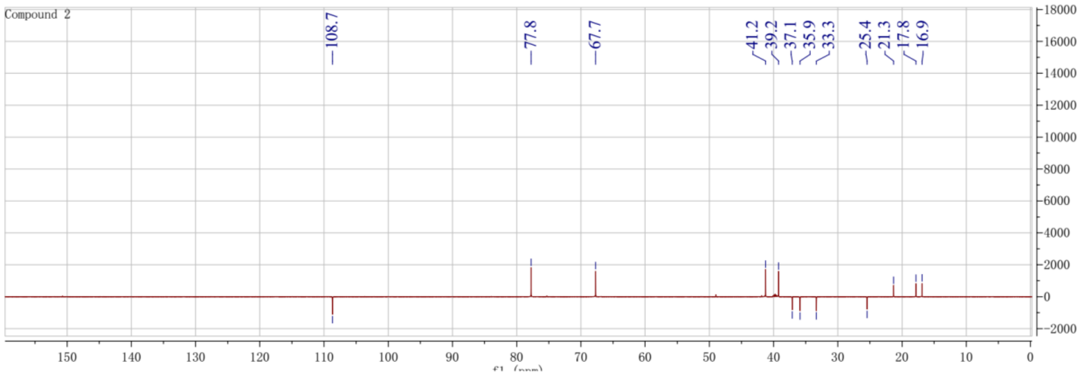

Figure S10**.** The ^{1}H-^{1}H COSY (500 MHz, DMSO-d_6) spectrum of compound **2**

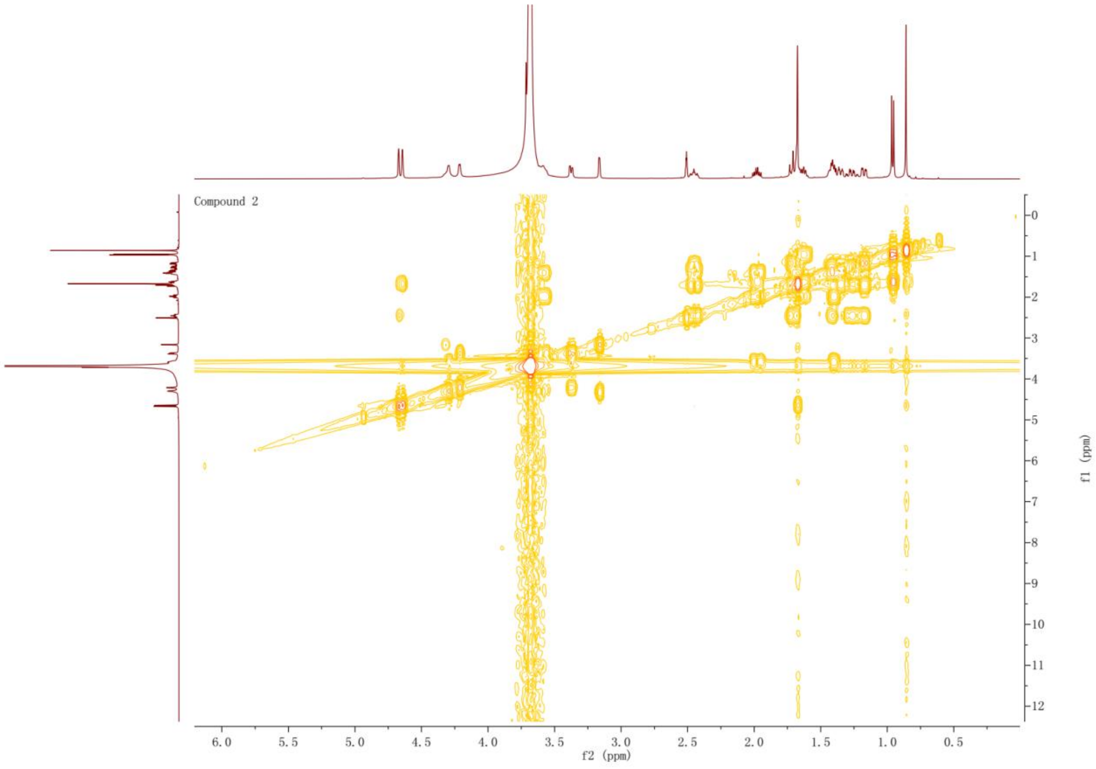

Figure S11. The HSQC (500 MHz, DMSO-d_6) spectrum of compound **2**

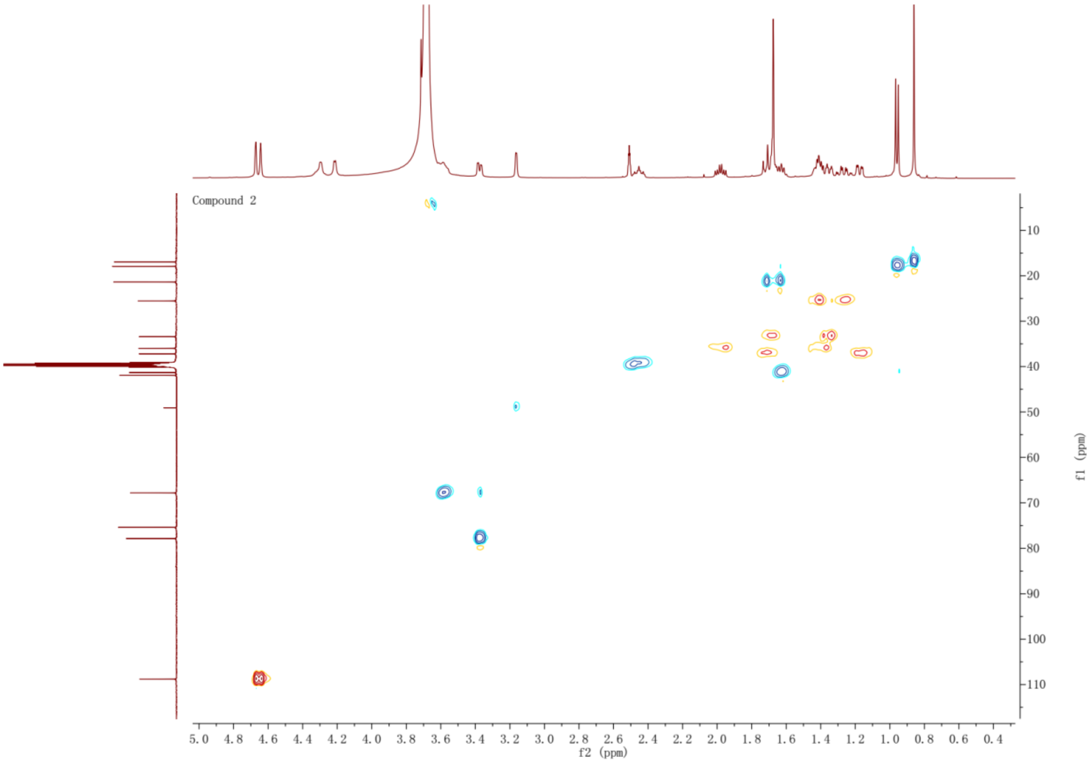

Figure S12. The HMBC (500 MHz, DMSO-d_6) spectrum of compound **2**

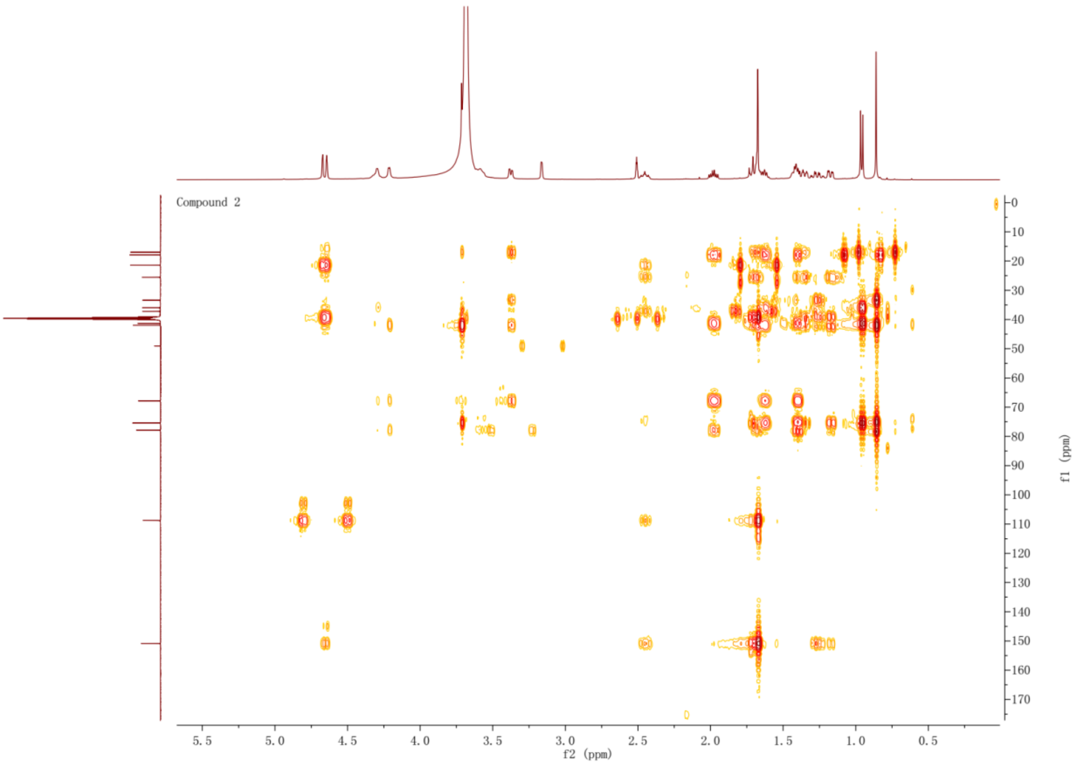

Figure S13. The ROESY (500 MHz, DMSO-d_6) spectrum of compound **2**

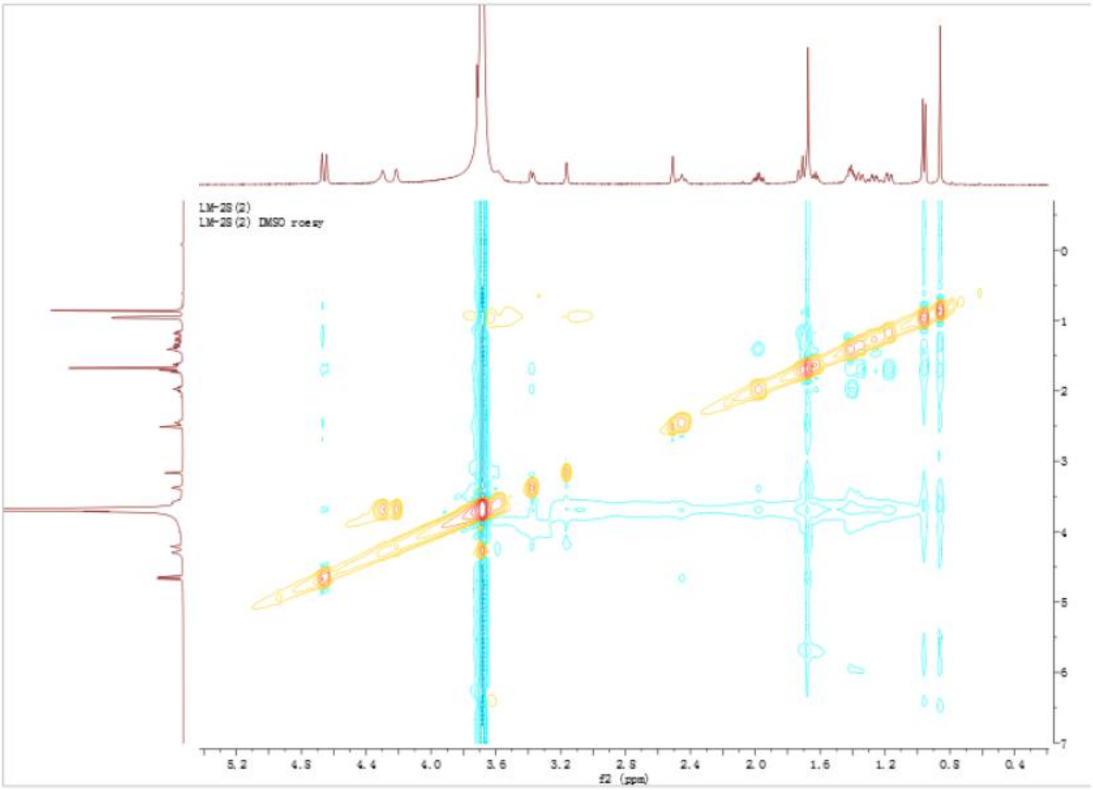

Figure S14. The HRESI spectrum of compound 2

Elemental Composition Report

Single Mass Analysis
Tolerance = 22.0 mDa / DBE: min = -10.0, max = 120.0
Selected filters: None

Monoisotopic Mass, Odd and Even Electron Ions
14 formula(e) evaluated with 1 results within limits (up to 51 closest results for each mass)
Elements Used:
C: 0-200 H: 0-400 O: 2-4

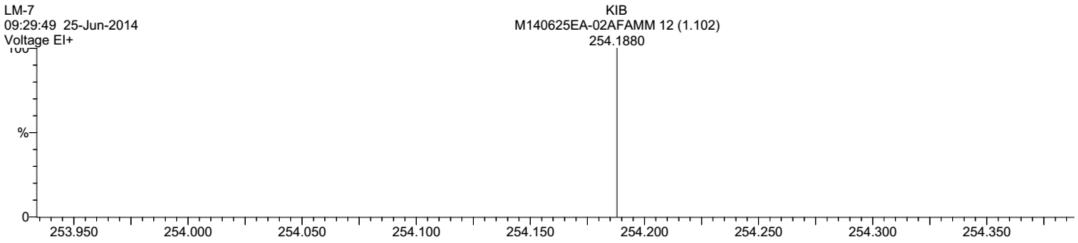

Minimum:				-10.0		
Maximum:		22.0	10.0	120.0		
Mass	Calc. Mass	mDa	PPM	DBE	i-FIT	Formula
254.1880	254.1882	-0.2	-0.8	3.0	5546025.5	C15 H26 O3

Figure S15.The ^{1}H NMR (500 MHz, DMSO-d_6) spectrum of compound **3**

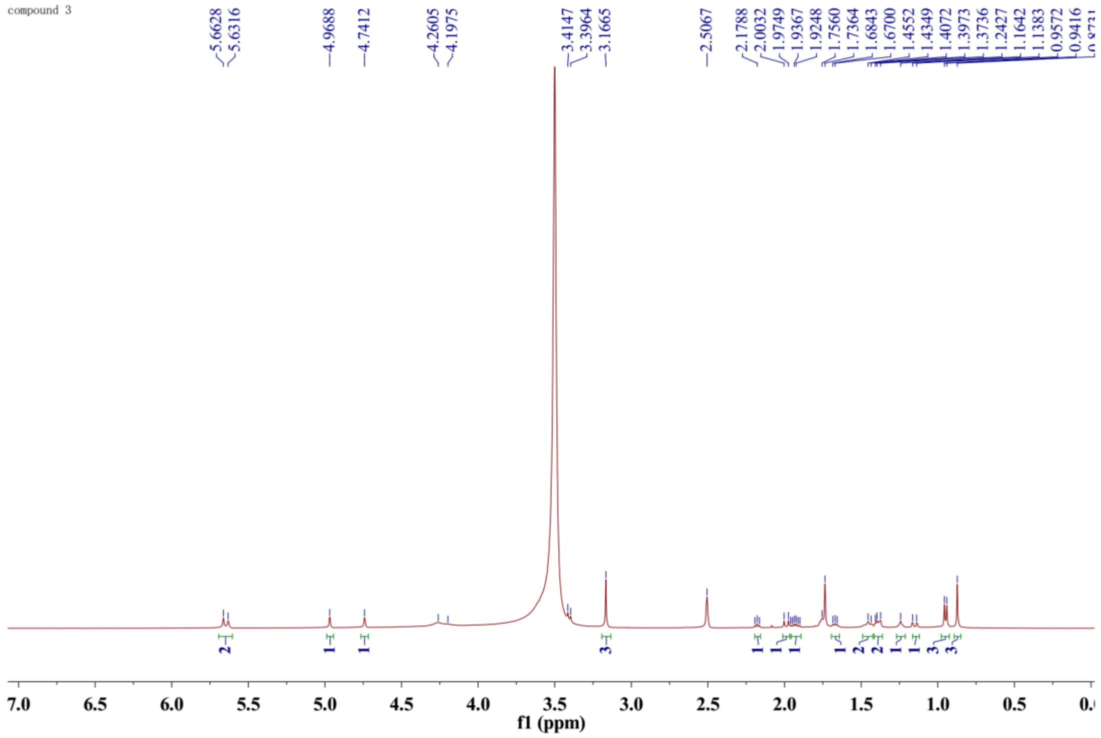

Figure S16. The ^{13}CNMR and DEPT135 (125 MHz, DMSO-d_6) spectrum of compound **3**

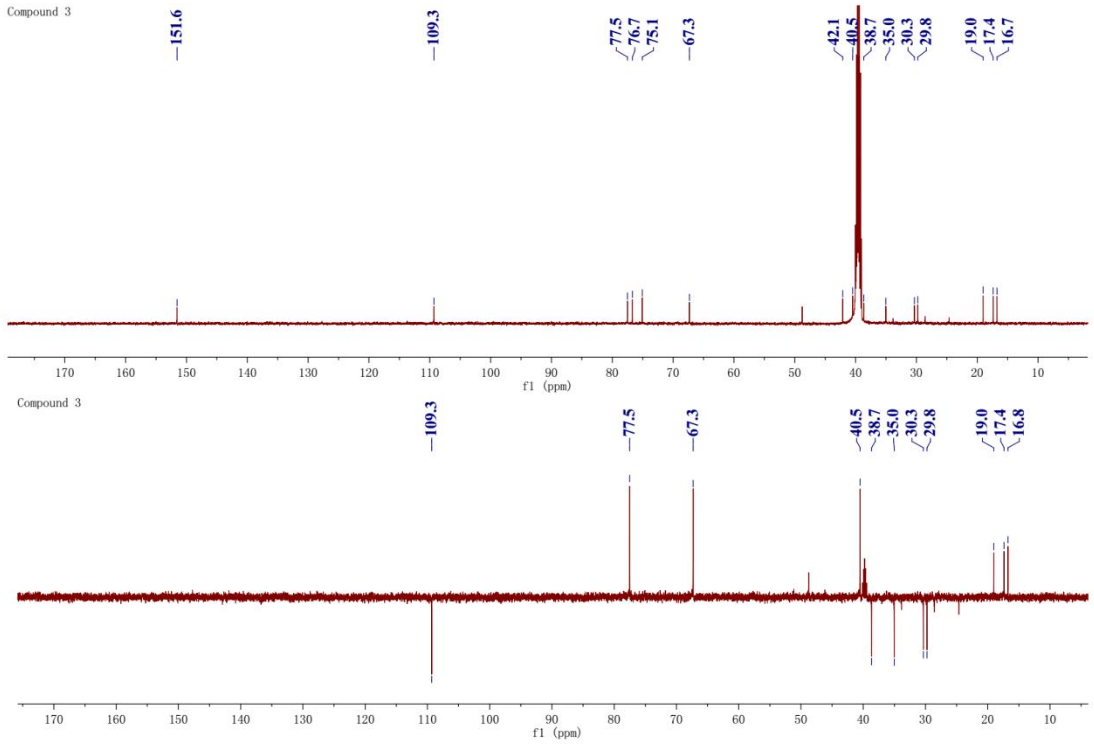

Figure S17. The ^{1}H-^{1}H COSY (500 MHz, DMSO-d_6) spectrum of compound **3**

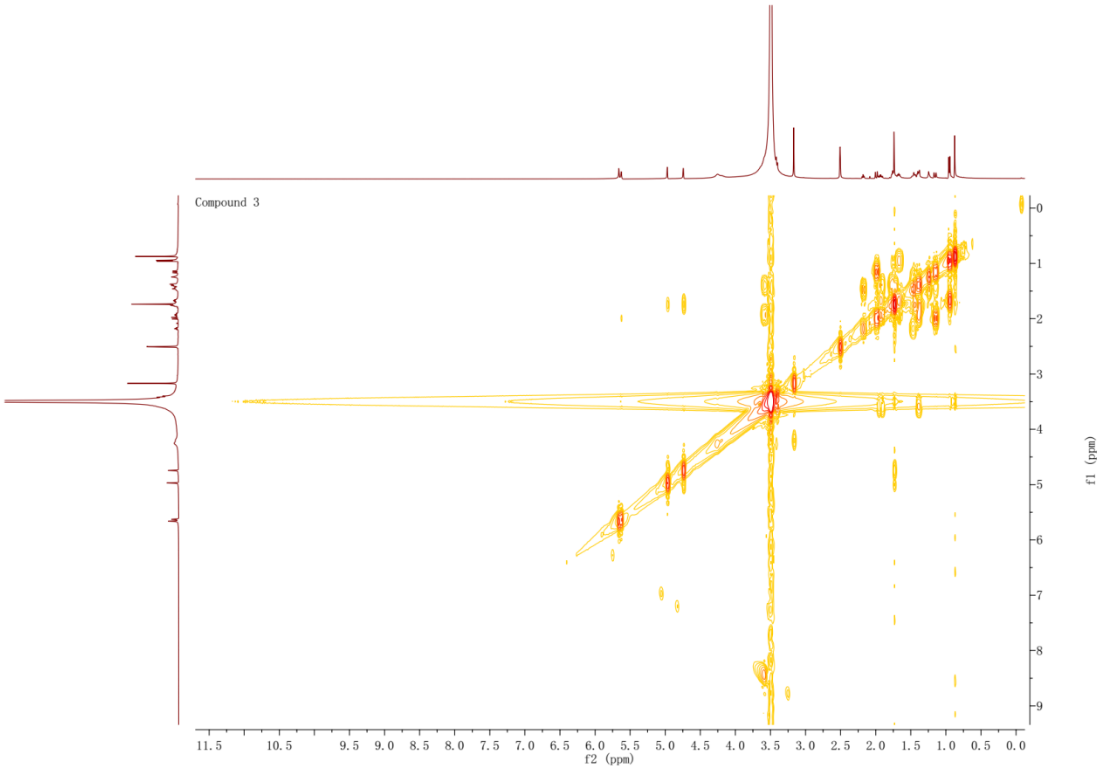

Figure S18. The HSQC (500 MHz, DMSO-d_6) spectrum of compound **3**

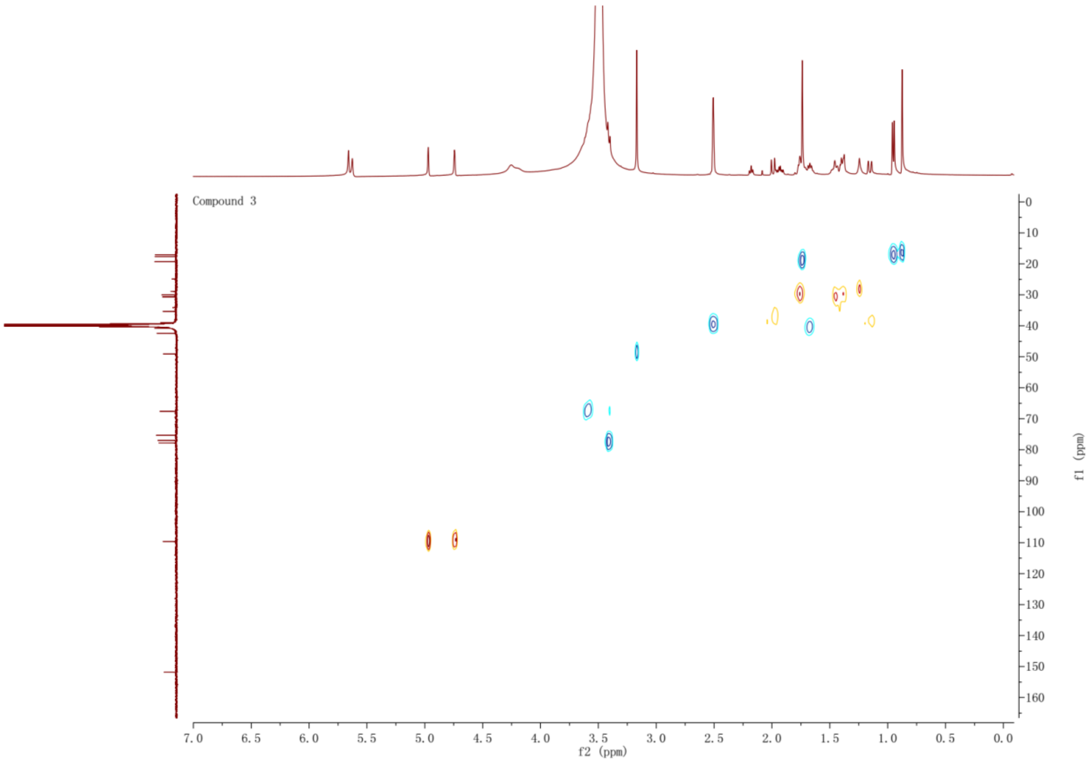

Figure S19. The HMBC (500 MHz, DMSO-d_6) spectrum of compound **3**

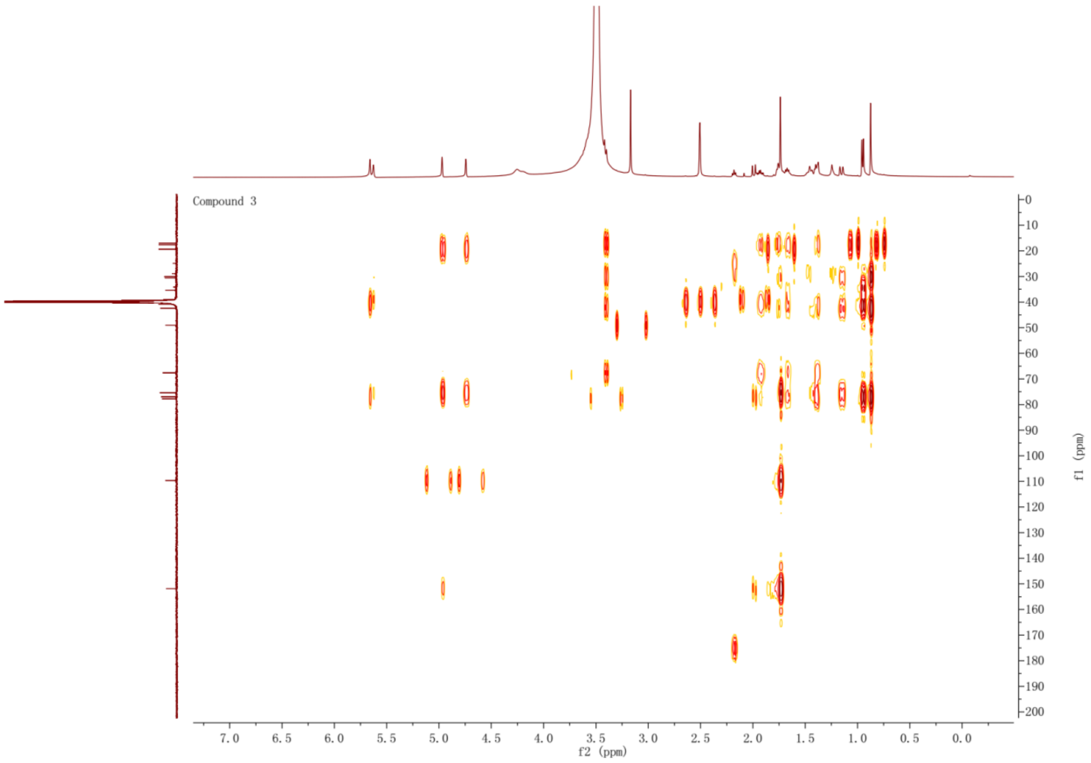

Figure S20. The ^{1}H-^{1}H ROESY (500 MHz, DMSO-d_6) spectrum of compound **3**

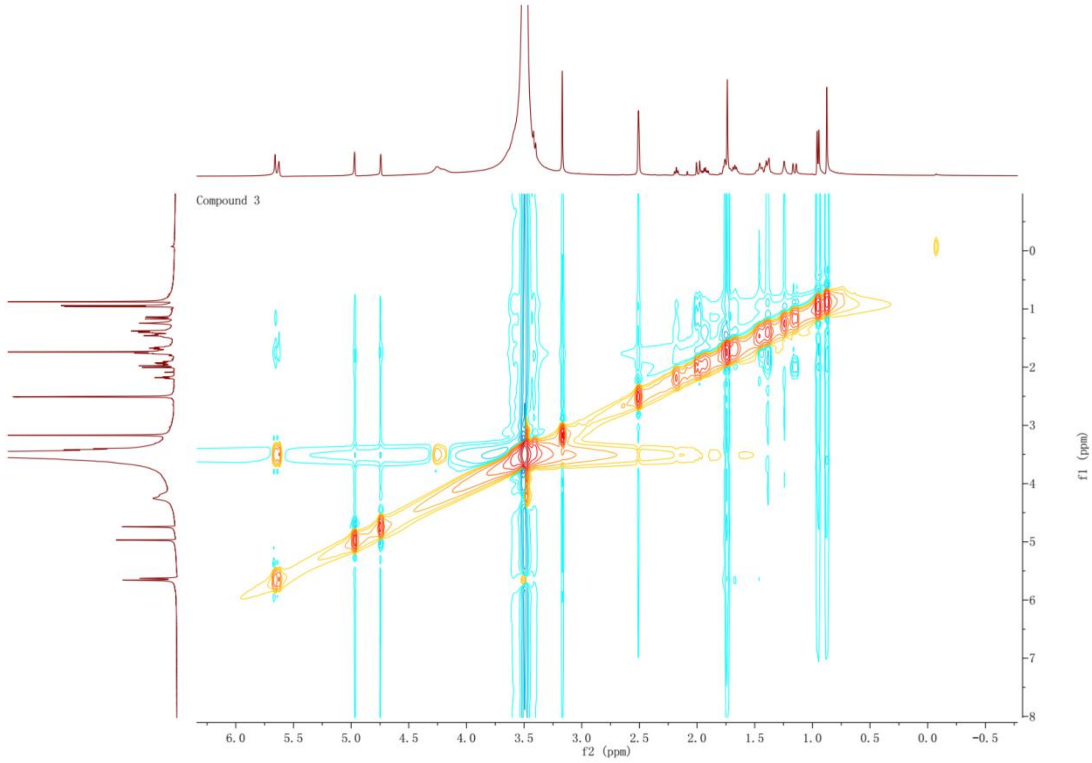

Figure S21. The HRESI spectrum of compound **3**

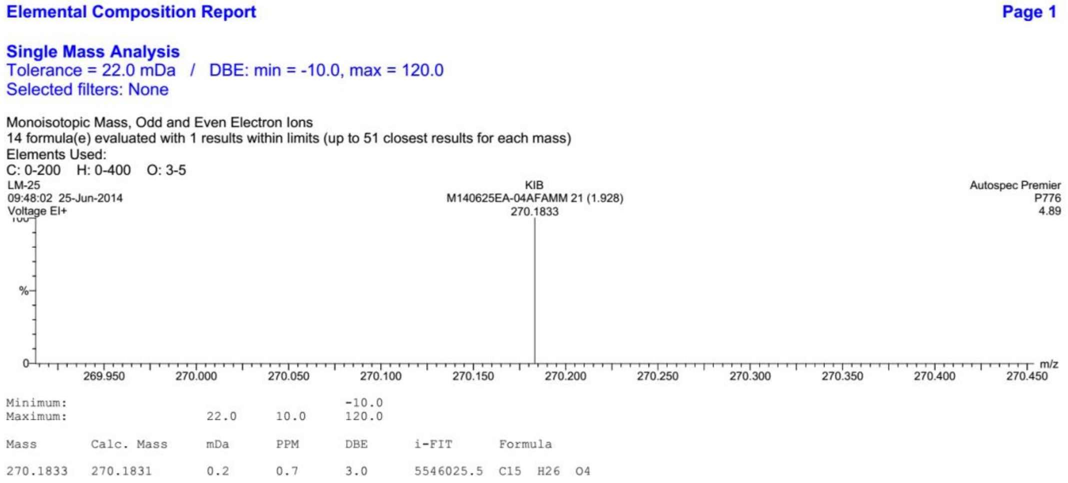

Figure S22 The ^{1}H NMR (500 MHz, DMSO-d_6) spectrum of compound **4**

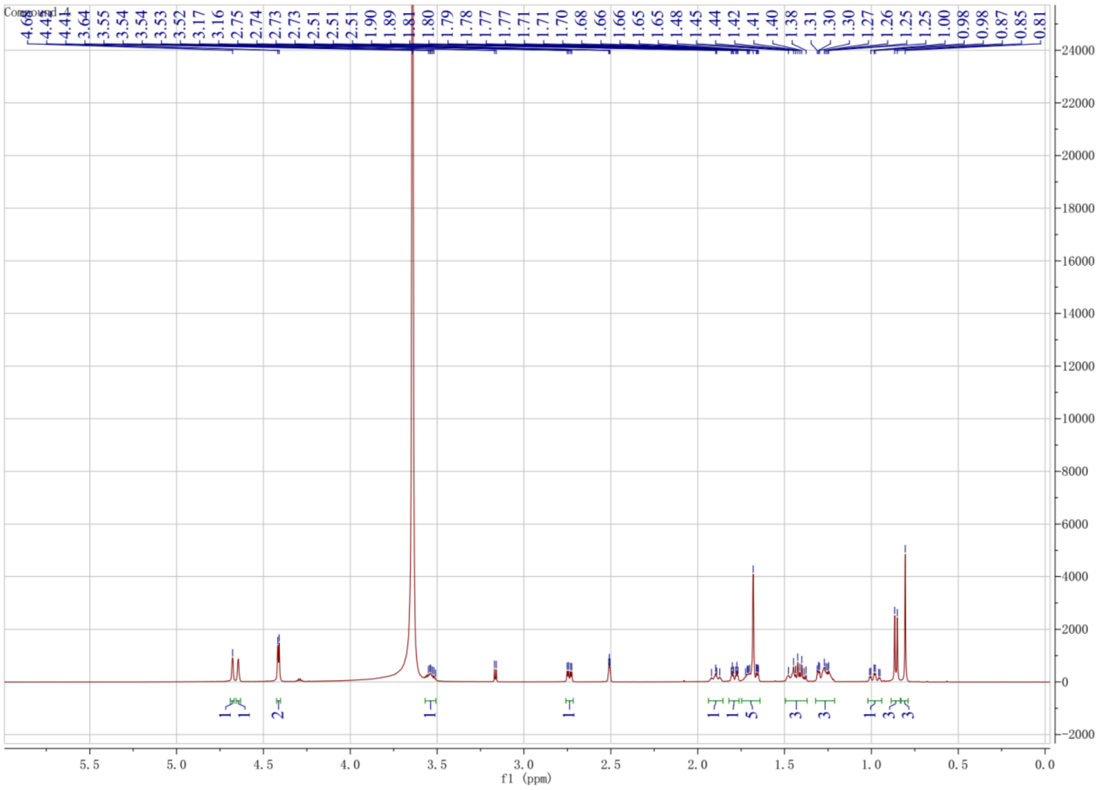

Figure S23. The ^{13}C NMR (125 MHz, DMSO-d_6) spectrum of compound **4**

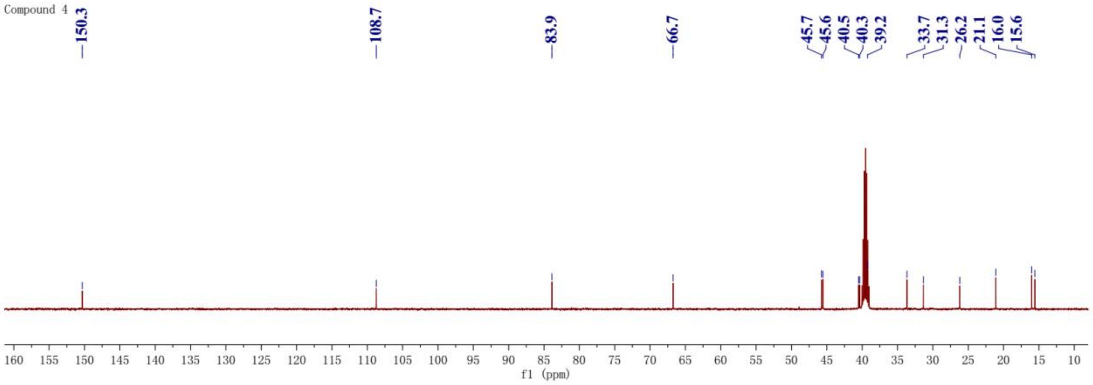

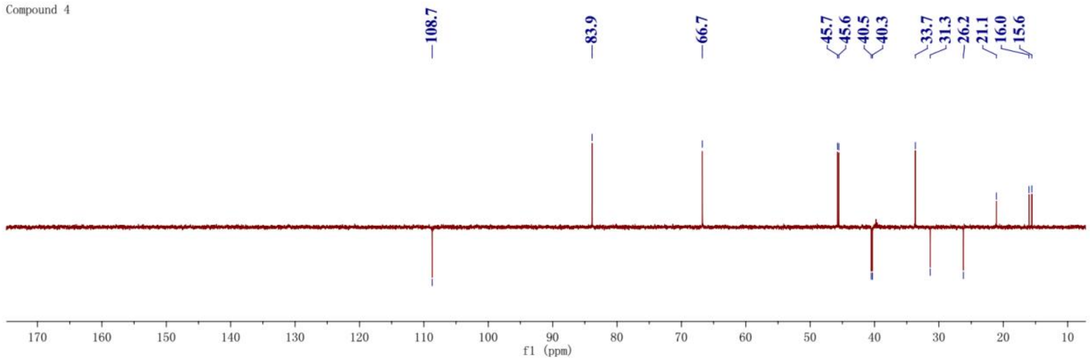

Figure S24. The ^{1}H-^{1}H COSY (500 MHz, DMSO-d_6) spectrum of compound **4**

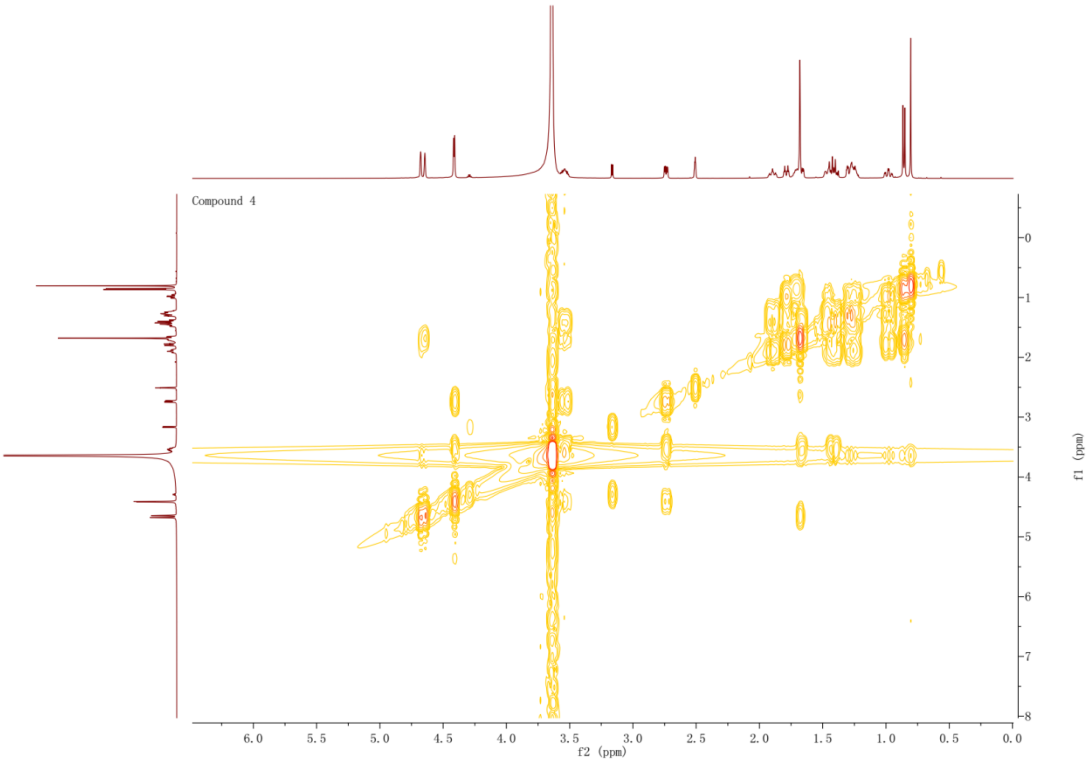

Figure S25. The HSQC (500 MHz, DMSO-d_6) spectrum of compound **4**

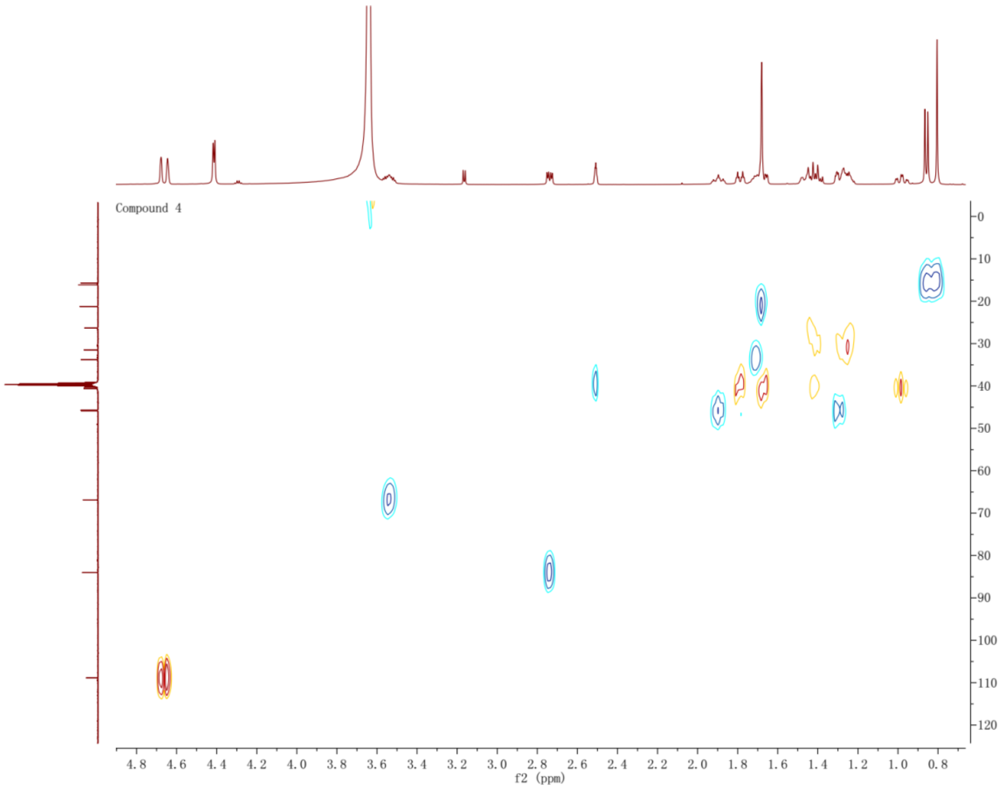

Figure S26. The HMBC (500 MHz, DMSO-d_6) spectrum of compound **4**

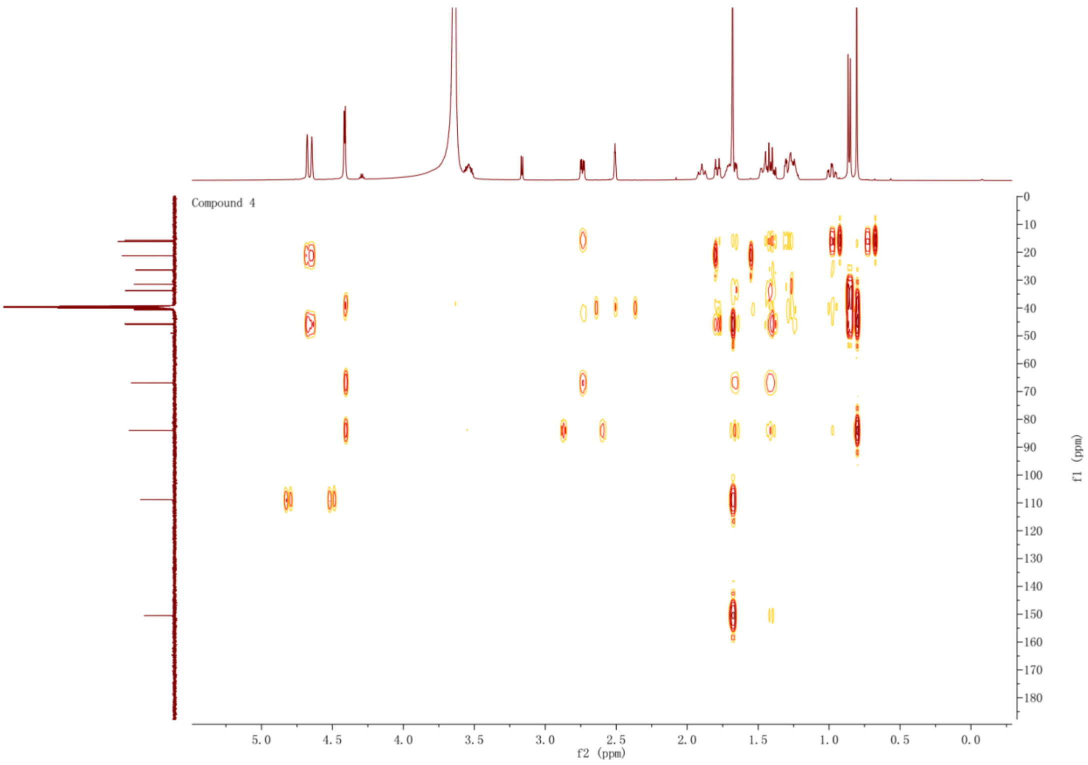

Figure S27. The ^{1}H-^{1}H ROESY (500 MHz, DMSO-d_6) spectrum of compound **4**

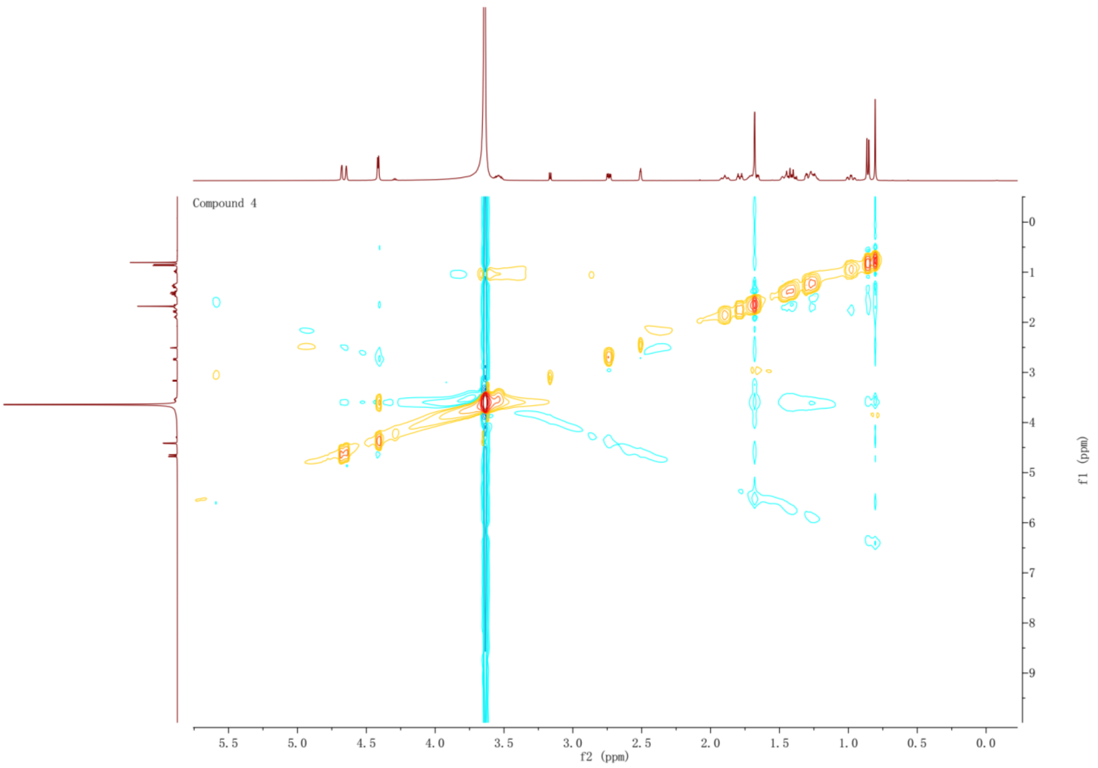

Figure S28. The HRESI spectrum of compound **4**

Elemental Composition Report **Page 1**

Single Mass Analysis
Tolerance = 22.0 mDa / DBE: min = -10.0, max = 120.0
Selected filters: None

Monoisotopic Mass, Odd and Even Electron Ions
14 formula(e) evaluated with 1 results within limits (up to 51 closest results for each mass)
Elements Used:
C: 0-200 H: 0-400 O: 1-3

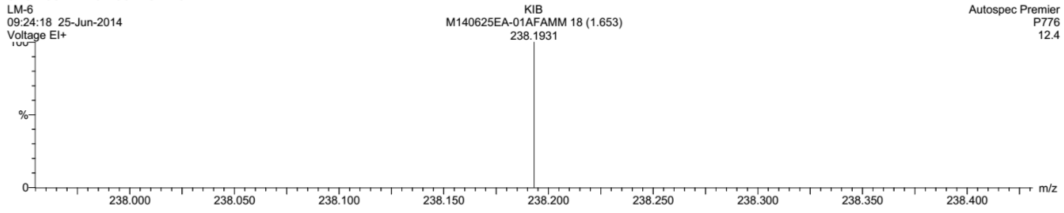

Minimum:				-10.0		
Maximum:		22.0	10.0	120.0		
Mass	Calc. Mass	mDa	PPM	DBE	i-FIT	Formula
238.1931	238.1933	-0.2	-0.8	3.0	5546026.0	C15 H26 O2

Figure S29. The ^{1}H NMR (500 MHz, CD_3OD-d_4) spectrum of *S*-MTPA ester **1a** and

R-MTPA ester **1b**

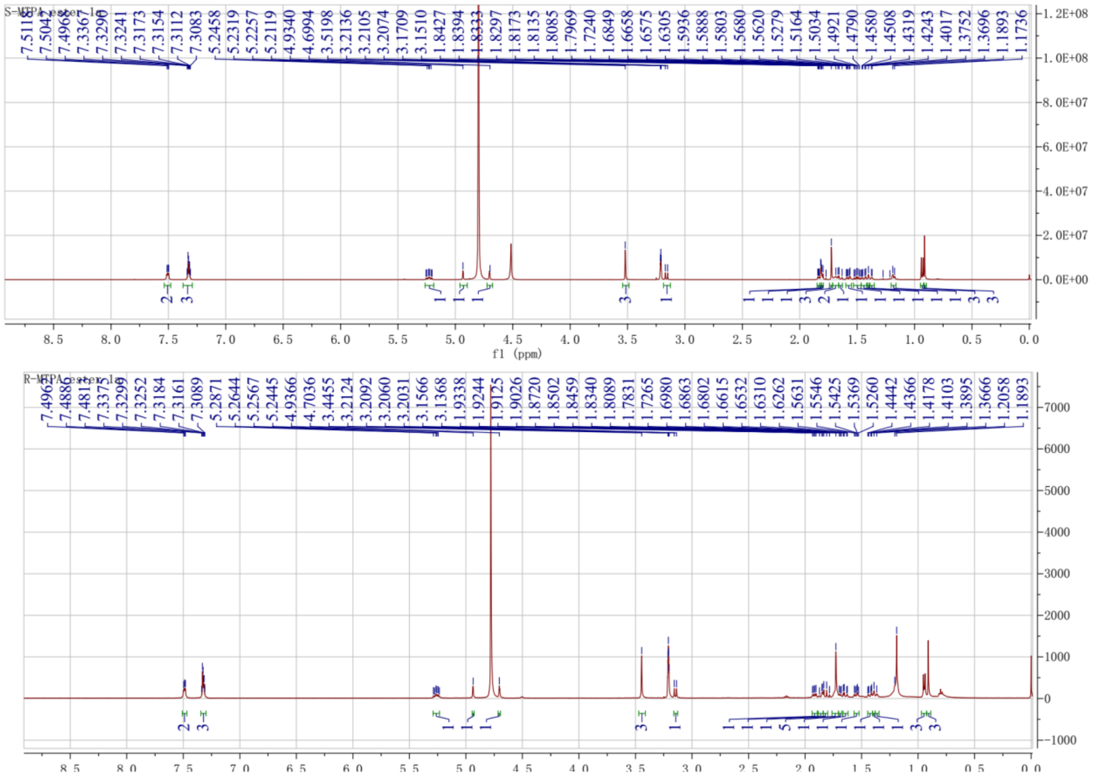

Figure S30. The ^{1}H NMR (500 MHz, CD_3COCD_3-d_6) spectrum of *S*-MTPA ester **2a** and *R*-MTPA ester **2b**

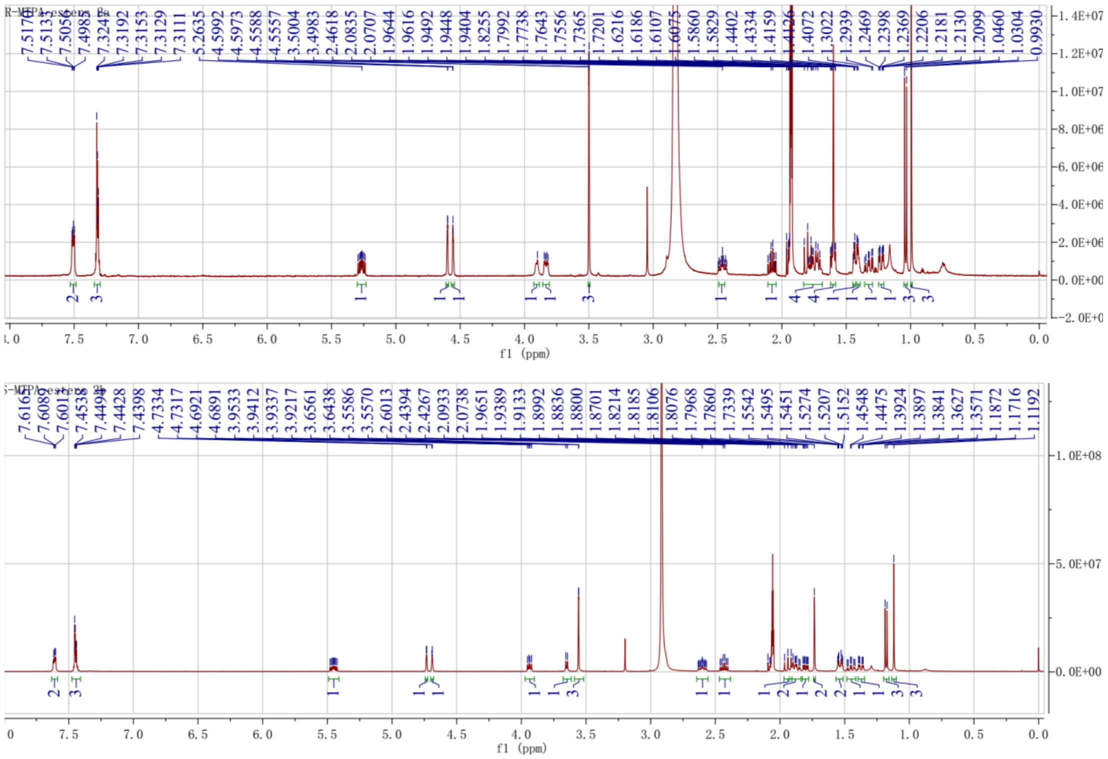

Figure S31. The ^{1}H NMR (500 MHz, CD_3OD-d_4) spectrum of *S*-MTPA ester **3a** and

R-MTPA ester**3b**

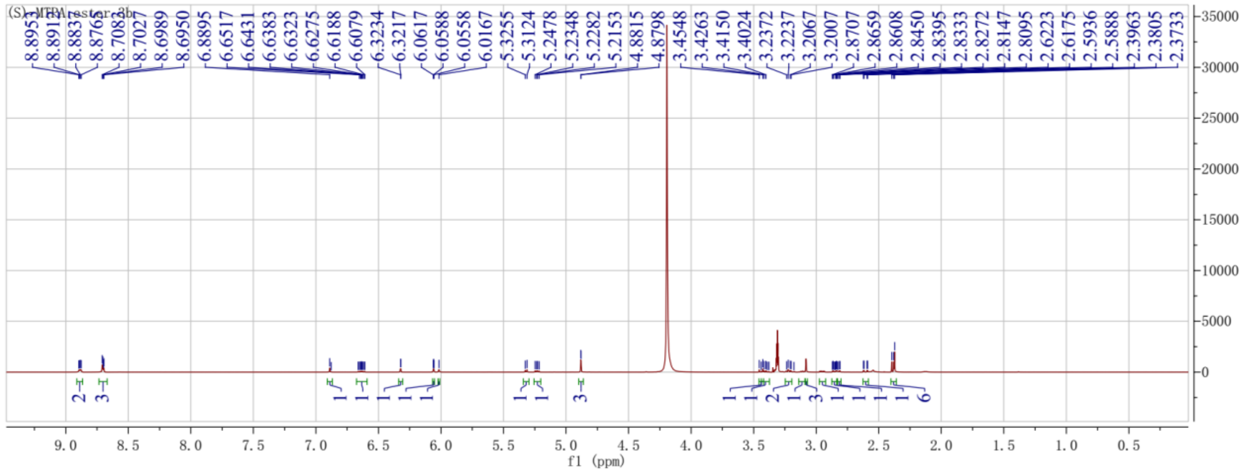

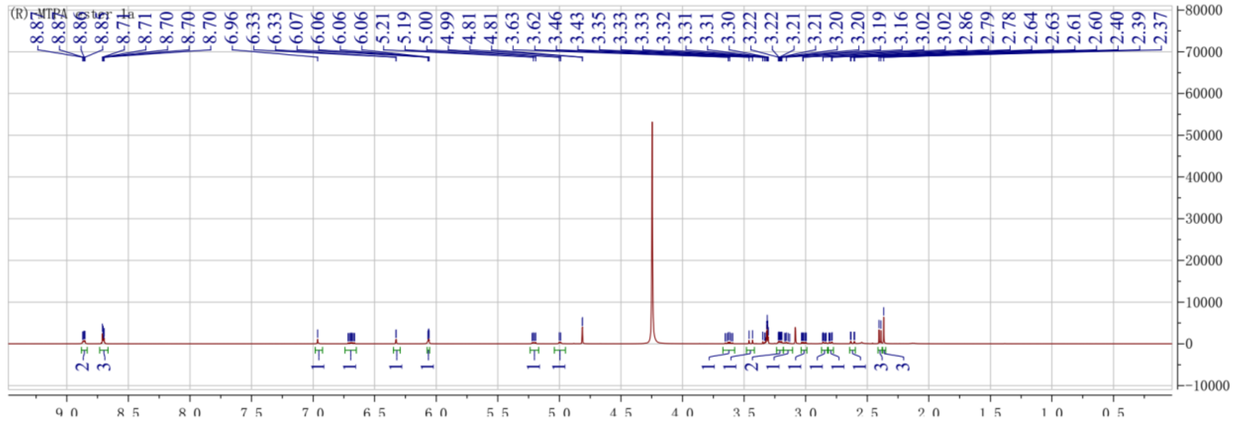

Supplement: Supplementary file 1 [file marinedrugs-16-00108-s001.zip › Supplementary Information.pdf]
